# Differential changes in cervical proprioception and motor imagery following structured imagery training in chronic neck pain

**DOI:** 10.1371/journal.pone.0361409

**Published:** 2026-11-05

**Authors:** Aysegul Bostan, Aysenur Akan, Ozlem Ulger

**Affiliations:** 1 Department of Physiotherapy and Rehabilitation, Faculty of Health Sciences, Bahcesehir University, Istanbul, Turkey; 2 Department of Physiotherapy and Rehabilitation, Faculty of Physical Therapy and Rehabilitation, Hacettepe University, Ankara, Turkey; 3 Neuroscience Research Center, Research Institute for Health Sciences and Technologies (SABITA), Istanbul Medipol University, Istanbul, Turkey; 4 Department of Neuroscience, Graduate School of Health Science, Istanbul Medipol University, Istanbul, Turkey; Ordu University, TÜRKIYE

## Abstract

**Background:**

Chronic neck pain (CNP) is associated with altered sensorimotor function, including impairments in cervical proprioception and changes in body-related perception. While implicit motor imagery (IMI)–based interventions have shown clinical benefits, their effects on cervical proprioceptive performance and motor imagery-related outcomes in individuals with CNP remain unclear.

**Objective:**

To investigate the effects of lateralization and IMI training on cervical proprioception (CP), neck awareness (NA), IMI performance, pain, disability, kinesiophobia, and range of motion (ROM) in individuals with CNP.

**Methods:**

In this clinical trial, 34 participants (17 CNP, 17 healthy controls) completed a 4-week structured imagery training (SIT) program combining lateralization and IMI training delivered via telerehabilitation. Primary outcome was cervical joint position error (JPE). Secondary outcomes included IMI performance (left/right judgment task), pain, disability, kinesiophobia, NA, ROM, and cognitive interference (Stroop).

**Results:**

Following SIT, the CNP group showed statistically significant within-group reductions in pain intensity (Holm-adjusted *p* = 0.012, *d* = 0.76), kinesiophobia (Holm-adjusted *p* = 0.034, *d* = 0.50), neck-related disability (Holm-adjusted *p* = 0.034, *d* = 0.37), and FreNAQ-T scores (Holm-adjusted *p* = 0.012, *d* = 0.09). However, baseline-adjusted models revealed no statistically significant between-group post-training differences for extension JPE (*p* > 0.05, partial η^2^ = 0.009), flexion JPE (where post-training values remained persistently elevated in CNP; partial η^2^ = 0.310), and IMI accuracy/reaction times for neck and hand tasks (all Holm-adjusted *p* = 1.000, partial η^2^ ≤ 0.068).

**Conclusions:**

A 4-week SIT program was associated with short-term improvements in subjective clinical symptoms in individuals with CNP; however, no differential group effects were observed for objective cervical proprioception or motor imagery performance.

## Introduction

CNP is a musculoskeletal problem that is prevalent worldwide and has significant negative effects on individuals’ daily activities, work performance, and quality of life [1,2]. This condition is not limited to pain alone; it involves multidimensional components such as proprioceptive impairments, movement control deficits, kinesiophobia, and changes in body perception [3–5]. Additionally, it has been reported that cognitive processes are associated with pain and disability in CNP, and that this condition supports the role of central mechanisms [6,7]. Current approaches have revealed that CNP is not merely a peripheral mechanical problem but is also associated with changes in sensory-motor integration and body representations at the central nervous system level [5,8–10].

Body schema is a dynamic neural representation formed by the integration of proprioceptive, visual, and vestibular inputs, playing a critical role in the planning and execution of movement [11,12]. This representation ensures that both real and imagined movements are correctly generated within biomechanical constraints [13,14]. Motor imagery is the process of mentally visualizing a movement without any physical movement occurring, and it shares largely common cortical and subcortical networks with the movements performed [15–17]. Therefore, motor imagery tasks are considered an indirect but powerful tool for assessing the functional integrity of the body schema [8].

One of the most widely used assessment methods for IMI is the left/right judgment task (LRJT) [9,18]. In this task, individuals are asked to decide whether body region images presented from different angles are on the right or left [9,13]. Prolonged reaction time reflects delayed access to the spatial representation of the body, while decreased accuracy reflects impairment in proprioceptive and cortical body representations [9,19]. There is consistent evidence that LRJT performance is impaired in individuals with chronic musculoskeletal pain, particularly in conditions affecting the extremities and face [9,17,20]. However, in chronic pain involving axial regions, particularly CNP, findings regarding LRJT performance are heterogeneous [9,21]. While some studies report impaired neck-specific laterality performance in individuals with CNP [5] others have found no significant impairment [20,22,23]. These inconsistencies have been suggested to stem from factors such as the duration of pain, the degree of chronicity, the specificity of the body region assessed, and the level of central adaptations [9]. It is thought that central reorganization and body schema changes may be more pronounced in individuals with long-term chronic pain [8].

Lateralization (left/right judgment) imagery training, which forms the first step of the graded motor imagery approach, is a non-invasive rehabilitation method that aims to reorganize body representations and improve sensory-motor integration [8,24,25]. Although lateralization training has been shown to have positive effects on pain and functional outcomes in extremity pain [17,26] studies that simultaneously address both central (motor imagery performance) and clinical (pain, proprioception, kinesiophobia, and NA) effects in CNP are limited. Furthermore, previous studies have generally examined motor imagery performance or clinical outcomes separately, rather than addressing both areas simultaneously. Despite growing interest in motor imagery–based interventions in chronic musculoskeletal pain, the relationship between peripheral sensorimotor impairments and central body representations remains insufficiently understood. Existing studies have predominantly focused either on clinical outcomes (e.g., pain and disability) or on motor imagery performance in isolation, often treating improvements in these domains as parallel processes. However, whether changes in proprioceptive accuracy are necessarily accompanied by changes in IMI performance remains unclear. This represents a critical gap, particularly in CNP, where findings regarding laterality performance are inconsistent and may reflect segment-specific or context-dependent adaptations rather than global impairments. Accordingly, there is a need for studies that simultaneously examine both peripheral and central components of the sensorimotor system to determine whether rehabilitation-induced changes occur in a coupled or dissociated manner. Addressing this question may provide important insights into the mechanisms underlying chronic pain and inform the development of more targeted rehabilitation strategies.

Therefore, this study aims to investigate the effects of a 4-week structured lateralization imagery training program implemented via telerehabilitation on both central and clinical outcomes, with a particular focus on proprioception as the primary outcome, and to compare these effects between individuals with CNP and healthy controls. It is hypothesized that this intervention will result in significant improvements in proprioception, as well as in secondary outcomes including LRJT performance (reaction time and accuracy), NA, pain intensity, and kinesiophobia, with greater improvements expected in individuals with CNP compared to healthy controls.

## Method

### Study design

This study used a prospective, non-randomized, two-group pre–post quasi-experimental design. Participants were included in either the CNP group or the HC group based on their clinical status and the relevant eligibility criteria; no random allocation was performed. Both groups received the same SIT program. The study was conducted between December 10, 2024, and November 4, 2025. Participant recruitment, group classification, follow-up, and analysis are presented in the participant flow diagram (Fig 1). Approval was obtained from Bahçeşehir University Clinical Research Ethics Committee with the number 2023-17/06 on 04/10/2023. The study was registered on ClinicalTrials.gov (NCT06679335). All participants provided informed consent before the start of this study.

**Fig 1:**
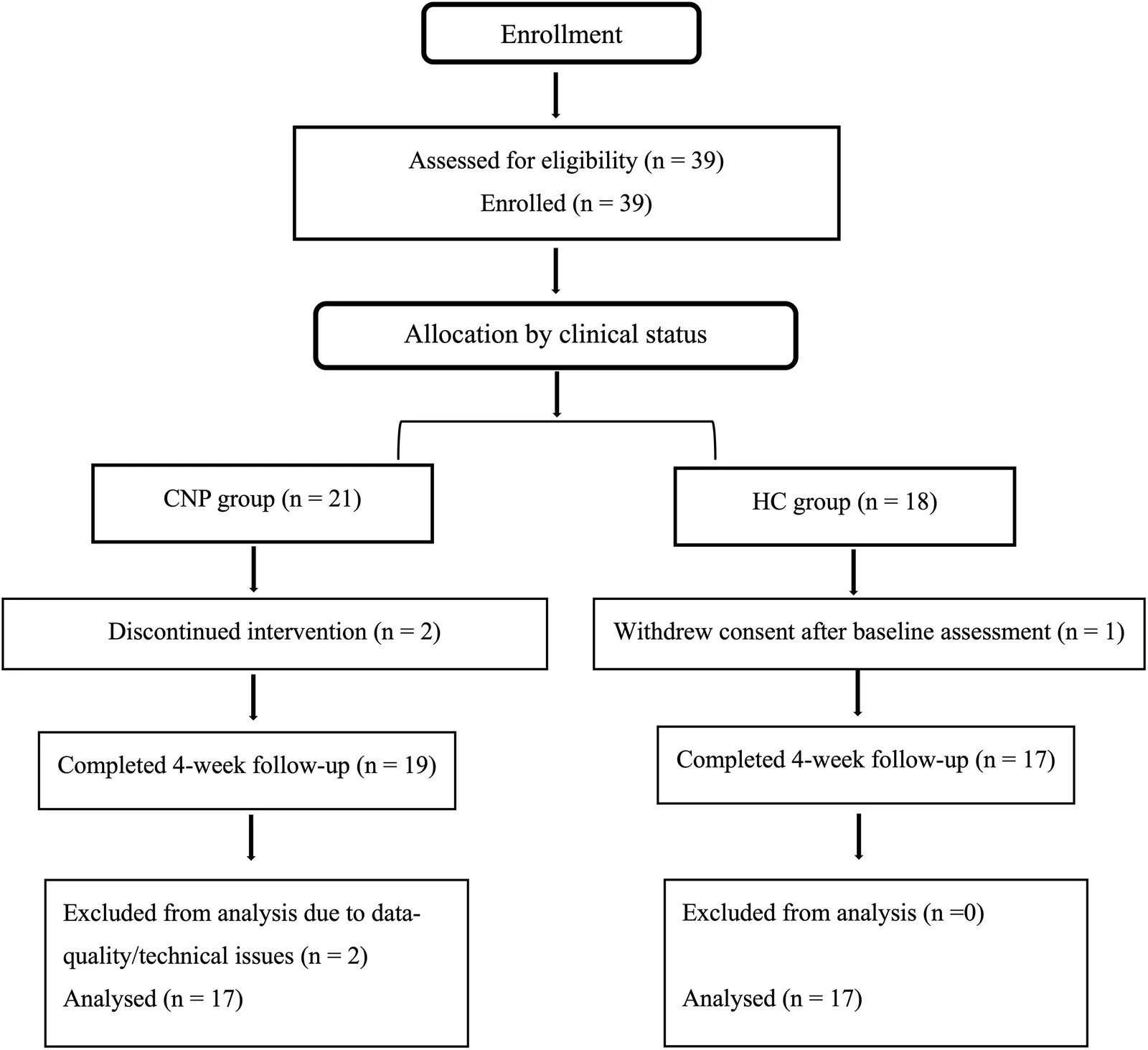
The participant flow diagram of the study.

### Participants and sample

The study included individuals with CNP who were clinically diagnosed by a physician and met prespecified eligibility criteria, and age- and sex-matched HC group. All participants were right-handed. Individuals eligible for inclusion in the CNP group were those who had neck pain for at least three months, were between 30 and 45 years of age, were able to take visual and cognitive assessments and follow the training protocol and had a MoCA score of 21 or higher. Participants in the HC group had no history of neck pain in the past year, were aged 30–45 years, and had a MoCA score of at least 21. Participants were excluded if they had a history of cervical spine surgery, neurological disorders (such as stroke, multiple sclerosis or peripheral neuropathy), vestibular dysfunction, rheumatologic or systemic inflammatory diseases, or severe psychiatric disorders that could affect visual perception, attention or motor imagery performance.

An a priori sample size calculation was performed using G*Power 3.1.9.2 for the group × time interaction in a two-group, two-time-point repeated-measures ANOVA (f = 0.25, α = 0.05, power = 0.80, repeated-measures correlation = 0.50, nonsphericity correction = 1.00). The required sample size was 34 participants (17 per group; actual power = 0.807). Supporting outcome-specific calculations were also conducted for cervical JPE using the within-group effect sizes reported by Sezerel and Yüksel [27] for flexion (*dz* = 0.81) and extension (*dz* = 0.89). These calculations indicated that 15 and 12 completers, respectively, were required; after allowing for 10% attrition, the more conservative flexion estimate supported a target of 17 participants per group. A total of 39 participants were enrolled. Five were not included in the final analysis: one participant in the HC group withdrew consent after the initial assessment, two participants with CNP discontinued the intervention, and two participants with CNP had non-analyzable data because of data-quality/technical issues. Recruitment continued until 34 participants with complete and analyzable data (17 per group) were obtained, and the final analyses were conducted using these 34 datasets (Fig 1).

### Intervention procedures

#### Lateralization and motor imagery training.

A SIT program was implemented for the individuals included in the study. The intervention program lasted 4 weeks and was conducted 3 days a week, for 30 minutes, via remote video conferencing with a physical therapist. Individuals in both groups were also asked to mentally visualize the learned exercises three times per week. The SIT program began with lateralization training and was applied for the first 2 weeks and every day a week. At this stage, lateralization training was performed using the “Recognise™ Neck and Hand” applications (Noigroup, Australia) [25,28], which were installed on participants’ smartphones (https://www.noigroup.com/). The task was administered using a touchscreen interface, and participants were familiarized with the application prior to data collection. Each session included an equal number of left and right images (20 images) presented in random order. The images were displayed at different rotation angles (0°, 90°, 180°, and 270°). Participants were instructed to respond as quickly and accurately as possible without focusing on their own body. Both accuracy and response time were automatically recorded by the application. The training was conducted during supervised sessions and continued at home. Participants were also encouraged to use the application independently outside supervised sessions.

Subsequently, the SIT continued with motor imagery training during weeks 3 and 4. Sessions were conducted in a quiet environment, and participants were instructed to focus entirely on the imagined movements without performing any physical action. Each session began with a brief body awareness phase, followed by imagined cervical movements within pain-free range (flexion, extension, lateral flexion, and rotation). The protocol then progressed to imagery of cervical stabilization exercises, including imagined isometric activations, craniocervical flexion (chin-tuck), and quadruped stabilization tasks such as cat–camel and alternating limb movements [29–32]. The imagery training included elements consistent with the principles of functional equivalence outlined in the PETTLEP (Physical, Environment, Task, Timing, Learning, Emotion, Perspective) model [33]. A summary of the lateralization and motor imagery training protocol is presented in Table 1, and detailed descriptions of the exercises are provided in S1 Appendix.

**Table 1:** Structured lateralization and motor imagery training protocol.

Week	Lateralization Training (Recognise™)	Motor Imagery Training
**Weeks 1–2**	Daily supervised and home-based lateralization training (7 days/week, total 14 sessions) using 20 standardized neck and hand images via the Recognise™ application. Participants performed right/left discrimination tasks without focusing on their own body.	—
**Weeks 3–4**	Daily home-based lateralization training was continued throughout the intervention period.	Supervised MI training (3 sessions/week, total 6 sessions, 30 min/session) delivered via telerehabilitation. **Components:** • Body awareness training (postural alignment and multisensory attention) • Motor imagery exercises (first-person perspective, pain-free, slow execution) **Exercise types:** • Cervical stretching (flexion, extension, lateral flexion) • Chin-tuck exercise • Deep cervical muscle activation (isometric contractions) • Stabilization exercises (theraband-assisted and quadruped tasks) Each movement was repeated 5–10 times (5–10 s per trial).

The interventions were administered by the same researcher. Participants’ compliance with the program was monitored through direct observation during each session, monitoring adherence to instructions and maintain attention.. Additionally, participants provided verbal feedback on imagery clarity and concentration at the end of each session.. Possible side effects (e.g., involuntary muscle spasms, teeth clenching, or neck tension) were queried and recorded after each session.

### Outcomes

Sociodemographic information was collected from all participants using a standardized sociodemographic data form. The same assessment protocol underwent the same assessment protocol pre-test and post-test the training, and all evaluations were conducted face-to-face. Each assessment session lasted approximately 45–60 minutes. To ensure standardization, assessments were performed in a fixed order: cognitive status, motor imagery performance, proprioception, and clinical outcome measures.

#### Proprioception assessment.

The primary outcome measure was CP, assessed using the Joint Position Error (JPE) test with a digital inclinometer (Dualer IQ; JTECH Medical, Salt Lake City, UT, USA). Participants were assessed while seated with their eyes closed. After being guided to a target position and asked to memorize it, they attempted to actively reposition to the same point. This procedure was repeated three times for flexion and extension movements. The absolute deviation from the target position (in degrees) was recorded as JPE, with lower values indicating better proprioceptive accuracy [34,35]. This method has demonstrated good to very good intra- and inter-rater reliability in both patients and healthy individuals [4,35].

#### Cognitive assessment.

Cognitive status was assessed using the MoCA and the Stroop Color-Word Test. The MoCA was used to determine eligibility [36,37] while the Stroop Test (Çapa version) evaluated executive functions, particularly selective attention and response inhibition [38]. The interference score (Stroop D; C–B difference) was used in analyses. MoCA was administered only at baseline, whereas the Stroop test was repeated post-intervention.

#### Assessment of motor imagery ability.

KVIQ was used to assess baseline motor imagery ability and intergroup homogeneity. Developed by Malouin et al. [39] and validated in Turkish by Dilek et al. [40] the 17-item questionnaire evaluates visual and kinesthetic imagery components, with higher scores indicating better imagery ability. The KVIQ was administered only at baseline.

#### Pain intensity.

Neck pain intensity was assessed using the visual analog scale (VAS). Participants were asked to rate their average neck pain over the past week on a scale from 0 (no pain) to 10 (unbearable pain) [41].

#### Neck disability index.

The level of functional impairment related to the neck was assessed using the neck disability index (NDI). The NDI is a valid and reliable measure for CNP, consisting of 10 items with a total score ranging from 0 to 50. Higher scores indicate greater functional disability [42].

#### Kinesiophobia.

The level of avoidance and fear of movement was assessed using the tampa kinesiophobia scale (TSK). High scores on the scale indicate a high level of kinesiophobia [43].

#### Body awareness.

Body awareness was assessed using the Fremantle Neck Awareness Questionnaire (FreNAQ-T) a validated and reliable body awareness scale adapted for the neck. High scores indicate impaired body perception [44,45].

#### Left/Right judgment task.

Implicit motor imagery performance was assessed using the LRJT, which involved images of the neck and hands. Participants were asked to decide as quickly and accurately as possible whether images presented from different angles and rotations were right or left. The main outputs obtained from this task were: Reaction time (ms) and accuracy rate (%) [24,46]. Hand images were used as a control task to assess general motor imagery capacity, while neck images were used to assess neck-specific body representations [25,28].

#### Experimental design and procedure.

A total of 48 stimuli (24 left and 24 right) were used for each condition and presented over two cycles, resulting in 96 trials for the neck images. In a separate session, the same procedure was applied for hand images. A rest period was provided between the two sessions. All images were presented at different rotation angles (0°, 90°, 180°, and 270°) in randomized order. The experimental paradigm was programmed using E-Prime software (Psychology Software Tools Inc., Pittsburgh, PA, USA, Fig 2). Prior to the experimental task, participants completed a short practice session to ensure that they understood the instructions. Reaction time (ms) and accuracy (%) were recorded automatically by the software.

**Fig 2:**
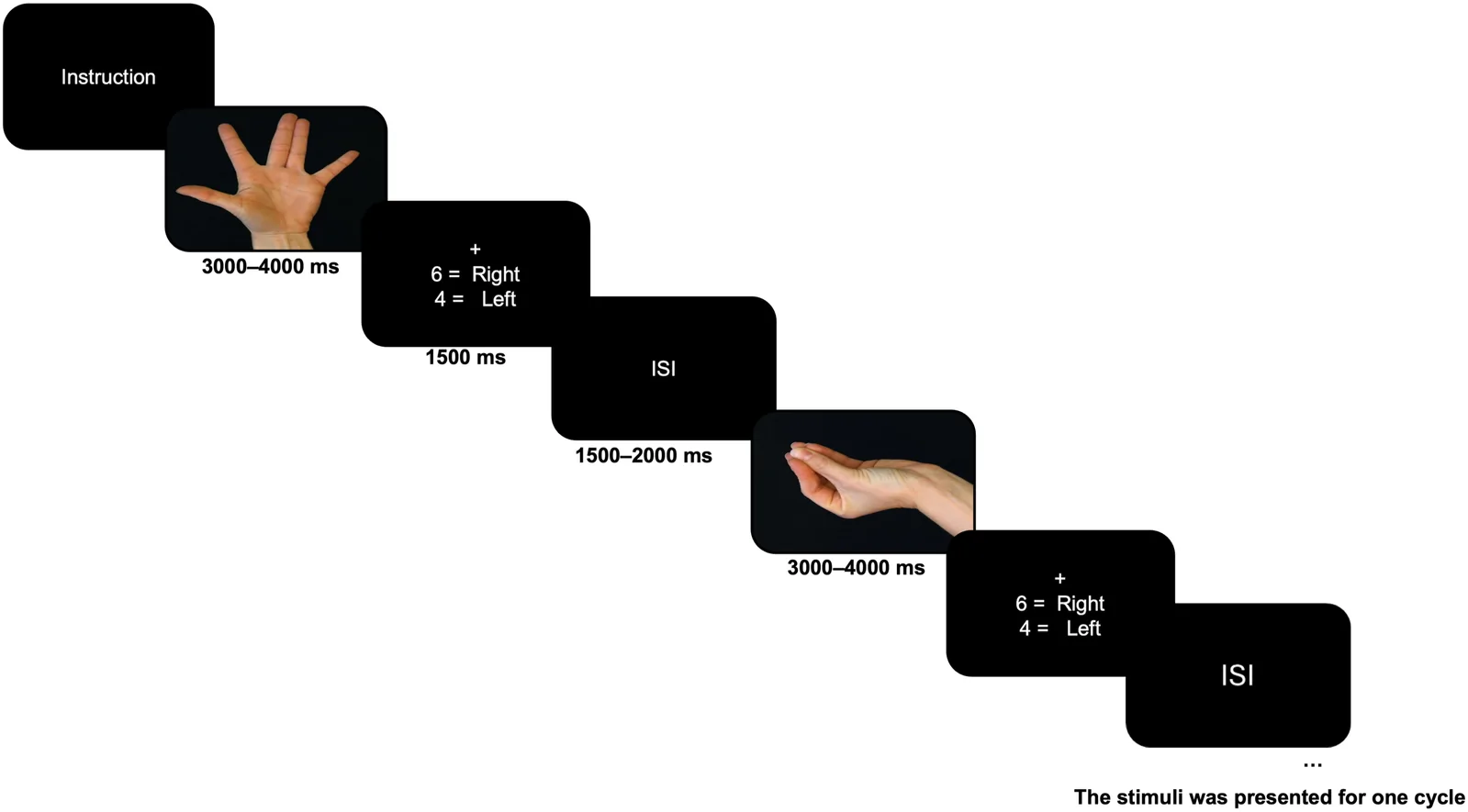
Experimental paradigm of the left/right judgment task illustrated using hand stimuli. The same procedure was applied to neck stimuli.

Each trial started with an instruction screen, followed by the presentation of a stimulus image for 3000–4000 ms. A fixation cross (+) was then displayed for 1500 ms, during which participants indicated whether the image corresponded to the right or left side by pressing the “6” key for right and the “4” key for left. This was followed by an inter-stimulus interval (1500–2000 ms), after which the next stimulus image was presented for 3000–4000 ms. The task was presented on a full-screen monitor (47.5 × 26.8 cm) positioned approximately 90 cm from the participants. Each session consisted of two cycles of the stimulus set [47].

### Statistics

All analyses were performed using IBM SPSS Statistics version 22.0 (IBM Corp., Armonk, NY, USA). Continuous variables were presented as mean ± standard deviation and range, and categorical variables as frequencies and percentages. Normality was assessed using the Shapiro–Wilk test. Baseline continuous variables were compared using independent-samples *t*-tests or Mann–Whitney *U* tests, as appropriate, while categorical variables were compared using two-sided Fisher’s exact tests. Within-group changes were examined using paired-samples *t*-tests or Wilcoxon signed-rank tests, as appropriate. Cohen’s *d* values were interpreted as small (0.20–0.49), medium (0.50–0.79), or large (≥0.80). Baseline balance was additionally evaluated using standardized mean differences (SMDs, Cohen’s *d* for continuous variables) and transformed Cramér’s *V* for categorical variables), with absolute values of <0.20, 0.20–0.50, and >0.50 indicating negligible, small, and moderate imbalance, respectively. Post-training outcomes were compared between groups using ANCOVA adjusted for the corresponding baseline value. Given the small sample, 5,000-replicate group-stratified BCa bootstrap analyses were used to obtain adjusted group means and CNP–HC differences with 95% confidence intervals. Partial eta squared (ηp^2^) was reported as the effect-size measure. To address multiplicity, the Holm step-down correction was applied separately within two inferential families: (1) the 16 baseline-adjusted between-group comparisons comprising the clinical, sensorimotor, cognitive, and Recognise laterality outcomes reported in Tables 6 and 8; and (2) the four within-group pre–post clinical comparisons evaluated in the CNP group (VAS, TSK, NDI, and FreNAQ-T) (Table 4). Both unadjusted and Holm-adjusted *p*-values were reported, and statistical significance was defined as a Holm-adjusted *p* < 0.05.

## Results

### Participants

The demographic characteristics of the participants are presented in Table 2. The HC (n = 17) and CNP (n = 17) groups were broadly comparable in terms of age, BMI, gender distribution, education level, smoking status, and alcohol consumption, with no statistically significant between-group differences (all *p* > 0.05). Absolute SMDs ranged from 0.000 to 0.463, indicating no large demographic differences between the groups. The gender distributions were identical (SMD = 0.000), while the largest standardized differences were observed for body mass index (SMD = 0.463) and age (SMD = 0.348).

**Table 2:** Demographic and clinical characteristics of participants at baseline.

Characteristic	HC Group (n = 17)	CNP Group (n = 17)	p	SMD
**Age (years)**, Mean ± SD (min–max)	36.29 ± 3.09 (30–41)	35.00 ± 4.24 (30–42)	0.259^a^	0.348
**BMI (kg/m^2^)**, Mean ± SD (min–max)	25.57 ± 4.04 (17–34)	27.90 ± 5.86 (18–43)	0.170^a^	0.462
**Gender**, n (%)			1.000^b^	0.000
Female	9 (52.9)	9 (52.9)		
Male	8 (47.1)	8 (47.1)		
**Pain Duration (years)**, n (%)			—	—
1–5 years	—	7 (41.2)		
5–10 years	—	1 (5.9)		
≥ 10 years	—	9 (52.9)		
**Education Level**, n (%)			1.000^b^	0.167
12 years	3 (17.6)	2 (11.8)		
≥ 16 years	14 (82.4)	15 (88.2)		
**Cigarette Use**, n (%)			1.000^b^	0.119
Yes	7 (41.17)	8 (47.05)		
No	10 (58.83)	9 (52.95)		
**Alcohol Use**, n (%)			0,656 ^b^	0.312
Yes	2 (11.76)	4 (23.52)		
No	15 (88.24)	13 (76.48)		

HC = healthy control; CNP = chronic neck pain; SD = standard deviation; BMI = body mass index; a = independent samples t-test; b = fisher’s exact test; SMD = standardized mean difference (Cohen’s *d*); values are presented as mean ± SD (minimum–maximum), and categorical variables as n (%).

Baseline MoCA, KVIQ, and Stroop D scores were broadly comparable between the groups, with no statistically significant differences (all *p* > 0.05; Table 3). Absolute SMDs indicated negligible differences in Stroop D (SMD = 0.027) and MoCA scores (SMD = 0.106), while a modest, standardized difference was observed in KVIQ scores (SMD = 0.337).

**Table 3:** Baseline cognitive and motor imagery measures in the HC and CNP groups.

Parameter	HC group (n = 17) Mean ± SD (min–max)	CNP group (n = 17) Mean ± SD (min–max)	*p* ^a^	SMD
**MoCA**	26.71 ± 1.89 (23–30)	26.47 ± 2.57 (21–30)	0.764	0.104
**KVIQ**	149.71 ± 18.31 (113–170)	143.18 ± 20.38 (112–170)	0.333	0.337
**Stroop D**	27.15 ± 8.005 (15.35–43.27)	27.40 ± 10.22 (9.98–51.53)	0.936	0.027

MoCA = Montreal Cognitive Assessment; KVIQ = Kinesthetic and Visual Imagery Questionnaire; a = independent-samples t-test; SMD = standardized mean difference (Cohen’s *d*); values are presented as mean ± SD (minimum–maximum).

### Clinical and sensorimotor findings

Clinical and sensorimotor findings are presented in Tables 4 and 5. In the CNP group, significant within-group reductions were observed after training in pain intensity (unadjusted *p* = 0.003; Holm-adjusted *p* = 0.012; Cohen’s *d* = 0.764), kinesiophobia (unadjusted *p* = 0.017; Holm-adjusted *p* = 0.034; Cohen’s *d* = 0.495), and neck-related disability (unadjusted *p* = 0.031; Holm-adjusted *p* = 0.034; Cohen’s *d* = 0.372) (Table 4). Baseline FreNAQ-T scores were substantially higher in the CNP group than in the HC group. After training, the CNP group showed a small reduction in FreNAQ-T scores, from 8.41 ± 4.66 to 8.00 ± 4.43 (mean change: −0.41; 95% CI: −0.67 to −0.15; unadjusted *p* = 0.004; Holm-adjusted *p* = 0.012), whereas the HC group showed no change. However, because of marked variance heterogeneity (Levene’s test, *p* < 0.001), a floor effect in the HC group, and limited overlap between the baseline distributions, a reliable baseline-adjusted model could not be fitted for FreNAQ-T. Therefore, FreNAQ-T was excluded from the primary ANCOVA framework, and its unadjusted within-group change is interpreted as an exploratory finding.

**Table 4:** Within-group and between-group comparisons of clinical and sensorimotor outcomes.

Parameters	CNP (n = 17) and HC (n = 17) Group	Pre-training	Post-training	Δ (post-pre) mean ± SD	Intra-group p	Pre-training Inter-group p	Post-training Inter-group p	Effect size Cohen’s d
**VAS, Mean ± SD**	CNP	6.82 ± 1.23	5.88 ± 1.23	−0.94 ± 1.09	**0.003**^**a**^ **(0.012)**			0.764
**TSK, Mean ± SD**	CNP	34.59 ± 2.16	33.53 _± 2.13_	−1.06 ± 1.64	**0.017**^**a**^ **(0.034)**			0.495
**NDI (%), Mean ± SD**	CNP	32.19 ± 3.58	30.86 ± 3.58	−1.33 ± 2.32	**0.031**^**a**^ **(0.034)**			0.372
**FreNAQ-T, Mean ± SD**	HC	0.65 ± 0.70	0.65 ± 0.70	**−**	**0.004**^**a**^ **(0.012)**		**<0.001** ^**b**^	0.121
	CNP	8.41 ± 4.66	8.00 ± 1.07	−0.41 ± 0.50				
**Stroop D, Mean ± SD**	HC	27.15 ± 8.05	25.05 ± 5.20	−2.10 ± 5.40	0.136	0.936	0.392	0.309
	CNP	27.40 ± 10.22	23.03 ± 8.07	−4.36 ± 8.34	**0.046** ^**a**^			0.474
**Cervical Flexion (º), Mean ± SD**	HC	49.55 ± 5.04	50.49 ± 4.60	0.94 ± 1.57	**0.026** ^**a**^	0.404	0.281	0.194
	CNP	47.76 ± 7.09	48.43 ± 6.23	0.66 ± 3.52	0.447			0.160
**Cervical Extension (º), Mean ± SD**	HC	48.98 ± 5.23	49.45 ± 5.23	0.47 ± 2.91	0.515	0.067	**0.040** ^**b**^	0.436
	CNP	44.27 ± 8.78	44.73 ± 7.42	0.45 ± 4.86	0.707			0.056
**Cervical LF Right (º), Mean ± SD**	HC	40.45 ± 4.40	41.33 ± 3.82	0.88 ± 4.57	0.438	0.762	0.538	0.213
	CNP	39.80 ± 7.54	40.18 ± 6.63	0.37 ± 3.75	0.688			0.053
**Cervical LF Left (º), Mean ± SD**	HC	40.49 ± 5.35	42.49 ± 4.80	2 ± 5.82	0.176	0.587	0.447	0.393
	CNP	39.29 ± 7.21	40.92 ± 6.88	1.62 ± 4.82	0.183			0.231
**Flexion JPE, Mean ± SD**	HC	2.47 ± 1.08	2.08 ± 0.72	−0.39 ± 1.10	0.163	**<0.001** ^**a**^	**<0.001** ^**b**^	0.424
	CNP	7.06 ± 4.14	6.67 ± 2.99	−0.39 ± 4.05	0.695			0.108
**Extension JPE, Mean ± SD**	HC	3.69 ± 1.73	3.67 ± 2.40	−0.1 ± 2.19	0.972	**0.015** ^**a**^	0.573	0.009
	CNP	5.77 ± 2.86	4.16 ± 2.60	−1.61 ± 2.95	**0.039** ^**a**^			0.589
**Sagittal plane (Flexion-Extension) JPE, Mean ± SD**	HC	3.08 ± 0.94	2.88 ± 1.22	−0.20 ± 1.07	0.450	**<0.001** ^**a**^	**<0.001** ^**b**^	0.183
	CNP	6.41 ± 3.18	5.41 ± 2.24	−1 ± 2.89	0.173			0.363

VAS = Visual Analog Scale; TSK = Tampa Scale for Kinesiophobia; NDI = Neck Disability Scale; FreNAQ-T = the Fremantle Neck Awareness Questionnaire; Stroop D = the resistance to interference score (C-B); JPE = joint position error; Mean = average; SD = standard deviation; a = Paired-samples t-test; b = Independent-samples t-test. Values in parentheses represent Holm-adjusted *p*-values calculated across the four within-group clinical outcomes in the CNP group (VAS, TSK, NDI, and FreNAQ-T). Statistical significance for these four outcomes was determined using the Holm-adjusted *p* < 0.05. All remaining within-group and between-group *p*-values in this table are unadjusted and are presented descriptively. Bold values indicate *p* < 0.05 according to the corresponding analysis.

**Table 5:** Between-group comparison of post–pre change scores in clinical and sensorimotor outcomes.

Parameters	HC Group Δmean ± SD	CNP Group Δmean ± SD	Between-group p (Δ)	Mean difference (95% CI)
**FreNAQ-T**	0.00 ± 0.00	−0.41 ± 0.50	0.004*	0.41 (0.15 to 0.67)
**Stroop D**	−2.10 ± 5.40	−4.36 ± 8.34	0.355	2.27 (−2.66 to 7.21)
**Cervical Flexion (º)**	0.94 ± 1.58	0.67 ± 3.53	0.771	0.27 (−1.63 to 2.18)
**Cervical Extension (º)**	0.47 ± 2.91	0.45 ± 4.87	0.989	0.02 (−2.78 to 2.82)
**Cervical LF Right (º)**	0.88 ± 4.57	0.37 ± 3.76	0.725	0.51 (−2.41 to 3.43)
**Cervical LF Left (º)**	2.00 ± 5.83	1.63 ± 4.82	0.840	0.37 (−3.36 to 4.11)
**Flexion JPE**	−0.39 ± 1.10	−0.39 ± 4.06	0.998	0.00 (−2.14 to 2.14)
**Extension JPE**	−0.02 ± 2.19	−1.61 ± 2.95	0.084	1.59 (−0.23 to 3.41)
**Sagittal plane JPE (Flexion-Extension)**	−0.20 ± 1.07	−1.00 ± 2.89	0.292	0.80 (−0.72 to 2.33)

HC = healthy control; CNP = chronic neck pain; CI = confidence interval; values are presented as mean ± SD. Change scores were calculated as post-training minus pre-training values. Between-group comparisons were performed using independent samples t-tests. Statistical significance was *p* < 0.05.

Regarding cervical proprioception, flexion JPE was significantly higher in the CNP group than in the HC group both at baseline (7.06 ± 4.14° vs. 2.47 ± 1.08°, unadjusted *p* < 0.001) and post training (6.67 ± 2.99° vs. 2.08 ± 0.72°, unadjusted *p* < 0.001) (Table 4). Both groups showed the same mean change (−0.39°), and the between-group difference in change scores was not statistically significant (mean difference = 0.00°, 95% CI: −2.14 to 2.14; *p* = 0.998; Table 5). Nevertheless, after adjustment for baseline flexion JPE, the CNP group continued to demonstrate significantly higher post-training values than the HC group (adjusted means: 6.030° vs. 2.715°; adjusted difference, CNP − HC = 3.316°, BCa 95% CI: 2.008 to 4.743; unadjusted bootstrap *p* = 0.001; Holm-adjusted *p* = 0.016; partial η^2^ = 0.310) (Table 6). These findings indicate that the pre-existing between-group difference in flexion JPE persisted post training, rather than reflecting a differential training response.

**Table 6:** Baseline-adjusted between-group comparisons of post-training clinical, sensorimotor, and cognitive outcomes.

Outcome	HC adjusted mean (BCa 95% CI)	CNP adjusted mean (BCa 95% CI)	Adjusted difference, CNP − HC (BCa 95% CI)	Unadjusted bootstrap *p*	Holm- adjusted p	Partial η²
**Stroop D**	25.118 (23.190–26.816)	22.976 (19.731–26.712)	−2.142 (−5.723–2.239)	0.261	1.000	0.043
**Flexion JPE (°)**	2.715 (1.879–3.623)	6.030 (4.903–7.210)	3.316 (2.008–4.743)	0.001	**0.016**	0.310
**Extension JPE (°)**	4.143 (3.002–5.360)	3.681 (2.699–4.727)	−0.462 (−2.041–1.069)	0.597	1.000	0.009
**Sagittal plane JPE (°)**	3.482 (2.674–4.316)	4.804 (4.023–5.640)	1.321 (0.346–2.410)	0.041	0.615	0.106
**Cervical flexion (°)**	49.779 (47.956–51.656)	49.143 (47.084–51.058)	−0.636 (−2.036–0.711)	0.438	1.000	0.017
**Cervical extension (°)**	47.709 (45.512–50.019)	46.468 (44.293–48.547)	−1.241 (−3.064–0.387)	0.275	1.000	0.028
**Cervical right lateral flexion (°)**	41.12 (39.13 to 43.25)	40.39 (37.89–42.81)	−0.73 (−3.39–1.58)	0.570	1.000	0.011
**Cervical left lateral flexion (°)**	42.14 (39.93 to 44.51)	41.27 (38.35–44.16)	−0.87 (−3.98–2.11)	0.600	1.000	0.009

HC = healthy control; CNP = chronic neck pain; BCa CI = bias-corrected and accelerated confidence interval. Adjusted means and between-group differences were estimated using ANCOVA controlling for baseline values. Confidence intervals and unadjusted *p*-values were obtained using 5,000-replicate group-stratified BCa bootstrap analyses. Holm-adjusted *p*-values were calculated jointly across the 16 baseline-adjusted comparisons reported in Tables 6 and 8. Partial η^2^ values were derived from the parametric ANCOVA models. Bold values indicate statistical significance based on Holm-adjusted *p* < 0.05.

Extension JPE decreased within the CNP group, from 5.77 ± 2.86° to 4.16 ± 2.60° (unadjusted *p* = 0.039; Cohen’s *d* = 0.589), whereas no significant change was observed in the HC group (3.69 ± 1.73° vs. 3.67 ± 2.40°; unadjusted *p* = 0.972; Cohen’s *d* = 0.009) (Table 4). Although the groups differed significantly at baseline (*p* = 0.015), no significant between-group difference was observed post training (*p* = 0.573). However, the between-group difference in change scores did not reach statistical significance (mean difference = 1.59°, 95% CI: −0.23 to 3.41; *p* = 0.084) (Table 5). Consistently, the baseline-adjusted analysis showed no significant post-training difference between the groups (adjusted means: 3.681° for CNP and 4.143° for HC; adjusted difference, CNP − HC=−0.462°, BCa 95% CI: −2.041 to 1.069; unadjusted bootstrap *p* = 0.597; Holm-adjusted *p* = 1.000; partial η^2^ = 0.009) (Table 6). Therefore, although extension JPE decreased within the CNP group, the between-group analyses did not demonstrate a differential response to training.

Regarding cervical ROM, no baseline differences were observed between groups (*p* > 0.05) (Table 4). For flexion ROM, a significant within-group increase occurred only in the HC group (from 49.55 ± 5.04° to 50.49 ± 4.60°; *t*(16) = −2.460, unadjus*t*ed *p* = 0.026; Cohen’s *d* = 0.194; CNP group: unadjusted *p* = 0.447) (Table 4), though neither change scores (mean difference = 0.27°, 95% CI: −1.63 to 2.18; *p* = 0.771) (Table 5) nor baseline-adjusted values (adjusted difference = −0.636°, BCa 95% CI: −2.036 to 0.711; Holm-adjusted *p* = 1.000; partial *η*^2^ = 0.017) (Table 6) differed significantly between groups.

For extension, right and left lateral flexion ROM, neither group demonstrated significant within-group changes (all unadjusted *p* > 0.05) (Table 4). Between-group change scores (Table 5) and baseline-adjusted post-training differences (extension: adjusted difference = −1.241°, BCa 95% CI: −3.064 to 0.387, Holm-adjusted *p* = 1.000; right LF: −0.73°, BCa 95% CI: −3.39 to 1.58, Holm-adjusted *p* = 1.000; left LF: −0.87°, BCa 95% CI: −3.98 to 2.11, Holm-adjusted *p* = 1.000) also showed no significant group differences (Table 6). Overall, training produced no differential effect on cervical ROM.

Regarding cognitive performance, the baseline-adjusted regression model for Stroop D scores revealed no statistically significant post-training difference between the CNP and HC groups (adjusted means: 22.976 for CNP vs. 25.118 for HC; adjusted difference = −2.142, BCa 95% CI: −5.723 to 2.239; Holm-adjusted p = 1.000) (Table 6). The small effect size (partial η^2^ = 0.043) confirms that the intervention yielded no differential training response between the groups.

### Neck and hand laterality findings

Descriptive statistics for hand and neck lateralization tasks, as well as unadjusted within-group and between-group comparisons, are presented in Table 7. Although changes in certain accuracy and reaction time measures were observed in the unadjusted within-group analyses, these findings were evaluated as exploratory results that were not corrected for multiple comparisons. ANCOVA models supported by 5,000-iteration, group-stratified BCa bootstrapping were applied to examine post-training between-group differences adjusted for baseline values (Table 8). After controlling for baseline values, no significant post-training difference was found between the CNP and HC groups in any of the accuracy and reaction time variables related to hand or neck lateralization (all bootstrap *p* > 0.05; all Holm-adjusted *p* = 1.000; partial η^2^ = 0.001–0.068).

**Table 7:** Descriptive statistics and unadjusted comparisons of accuracy and reaction time in the hand and neck laterality tasks.

Task/ Body Region	Images	Session	HC Group (Mean ± SD) (n = 17)	CNP Group (Mean ± SD) (n = 17)	Inter-group p^a^
**Neck Laterality**					
**Accuracy (%)**	**Right**	Pre-training	85.41 ± 10.28	83.82 ± 13.86	0.973
		Post-training	86.76 ± 9.98	87.13 ± 7.76	0.838
	Intra-group p^b^		0.917	0.591	
	**Left**	Pre-training	87.49 ± 9.79	86.76 ± 15.22	0.518
		Post-training	89.58 ± 9.80	89.70 ± 9.44	0.919
	Intra-group p^b^		0.333	0.681	
**RT (ms)**	**Right**	Pre-training	538.86 ± 151.80	475.56 ± 184.67	0.193
		Post-training	456.78 ± 135.59	448.00 ± 224.99	0.454
	Intra-group p^b^		**0.010**	0.193	
	**Left**	Pre-training	545.97 ± 147.23	481.66 ± 209.95	0.092
		Post-training	479.75 ± 151.29	475.26 ± 247.60	0.540
	Intra-group p^b^		**0.049**	0.435	
**Hand Laterality**					
**Accuracy (%)**	**Right**	Pre-training	78.52 ± 15.46	88.23 ± 17.11	**0.016** ^**a**^
		Post-training	83.82 ± 14.20	93.08 ± 9.07	**0.011** ^**a**^
	Intra-group p^b^		0.061	0.307	
	**Left**	Pre-training	79.85 ± 13.36	89.41 ± 14.51	**0.013** ^**a**^
		Post-training	84.70 ± 15.07	94.55 ± 2.11	**0.011** ^**a**^
	Intra-group p^b^		**0.026**	**0.045**	
**RT (ms)**	**Right**	Pre-training	604.83 ± 130.82	514.86 ± 181.70	0.107^d^
		Post-training	561.62 ± 182.50	523.43 ± 228.58	0.594^d^
	Intra-group p^**c**^		0.203	0.866	
	**Left**	Pre-training	626.51 ± 150.09	501.15 ± 179.18	**0.018** ^**a**^
		Post-training	567.00 ± 167.53	533.97 ± 237.70	0.643^d^
	Intra-group p^b^		0.112	0.906	

RT = reaction time; SD = standard deviation; HC = healthy control; CNP = chronic neck pain; a = Mann–Whitney U test; b = Wilcoxon signed-rank test; c = paired-samples t-test; d = independent-samples t-test. All *p*-values in this table are unadjusted and are presented descriptively. Bold values indicate unadjusted *p* < 0.05.

**Table 8:** Baseline-adjusted between-group comparisons of post-training accuracy and reaction time in the hand and neck laterality tasks.

Outcome	HC adjusted mean (BCa 95% CI)	CNP adjusted mean (BCa 95% CI)	Adjusted difference, CNP − HC (BCa 95% CI)	Unadjusted bootstrap *p*	Holm-adjusted *p*	Partial η²
**Neck right accuracy (%)**	86.421 (81.408–90.704)	87.476 (84.601–90.117)	1.055 (−2.986–5.128)	0.678	1.000	0.006
**Neck left accuracy (%)**	89.429 (83.908–93.610)	89.860 (86.351–93.185)	0.431 (−3.919–5.065)	0.884	1.000	0.001
**Neck right RT (ms)**	431.775 (372.747–499.931)	473.006 (390.048–566.368)	41.231 (−45.694–133.601)	0.448	1.000	0.025
**Neck left RT (ms)**	451.403 (387.575–531.772)	503.615 (420.138–590.211)	52.213 (−29.078–127.333)	0.302	1.000	0.041
**Hand right accuracy (%)**	85.670 (79.670–91.860)	91.240 (85.790–94.780)	5.560 (−2.050–10.920)	0.178	1.000	0.068
**Hand left accuracy (%)**	87.030 (79.530–94.150)	92.240 (86.680–95.700)	5.210 (−2.400–10.100)	0.293	1.000	0.057
**Hand right RT (ms)**	527.730 (446.470–608.630)	557.330 (468.660–660.170)	29.600 (−83.970–157.060)	0.654	1.000	0.007
**Hand left RT (ms)**	535.240 (460.700–606.370)	565.730 (471.550–670.670)	30.490 (−86.560–164.400)	0.642	1.000	0.006

HC = healthy control; CNP = chronic neck pain; BCa CI = bias-corrected and accelerated confidence interval; RT = reaction time. Adjusted means and between-group differences were estimated using ANCOVA controlling for baseline values. Confidence intervals and unadjusted *p*-values were obtained using 5,000-replicate group-stratified BCa bootstrap analyses. Holm-adjusted *p*-values were calculated jointly across the 16 baseline-adjusted comparisons reported in Tables 6 and 8. Partial η^2^ values were derived from the parametric ANCOVA models. Bold values indicate statistical significance based on Holm-adjusted *p* < 0.05.

## Discussion

This study compared the changes in clinical, sensorimotor, and cognitive outcomes observed in participants with CNP and healthy participants who received the same structured lateralization and motor imagery training. In the CNP group, statistically significant but modest improvements were observed in pain intensity, kinesiophobia, neck-related disability, and FreNAQ-T scores following the training. In contrast, objective sensorimotor and cognitive measures showed no consistent differences in response to training between groups. Although the cervical extension JPE decreased after training in the CNP group, this change did not differ significantly from that observed in the healthy control group; nor were there any differences between the groups after training compared to baseline values. Cervical flexion JPE did not change significantly within either group, and the change scores did not differ significantly between the groups. However, after adjustment for baseline values and multiple comparisons, post-training cervical flexion JPE remained significantly higher in the CNP group than in the HC group. Similarly, measurements of accuracy and reaction time on lateralization tasks and cervical ROM also showed no difference in the training response between the groups. Overall, these findings suggest that the reductions observed in some subjective clinical outcomes in the CNP group were not accompanied by corresponding improvements in objective sensorimotor and cognitive performance. Furthermore, the between-group difference in proprioceptive performance, particularly in cervical flexion JPE, persisted after training.

Consistent with previous studies, reductions in pain intensity, neck-related disability, and kinesiophobia were observed in the CNP group after training, and these changes remained statistically significant after Holm correction [48,49]. However, the magnitude of the changes in VAS, NDI, and FreNAQ-T scores was relatively small. Therefore, their clinical relevance should be interpreted cautiously and confirmed in future controlled studies using established minimal clinically important difference thresholds. It has previously been reported that education-based interventions and motor imagery approaches may have positive effects on processes related to pain perception, fear of movement, and pain-related functional limitations [49,50]. However, the absence of a non-intervention CNP control group in the present study limits the extent to which the observed clinical improvements can be attributed directly to SIT. Therefore, it cannot be determined to what extent these changes are distinguishable from the natural progression of the condition, effects associated with repeated measurements, participant expectations, or nonspecific effects related to participation in the intervention. Within this context, the findings show that clinical improvements were observed after SIT; however, they do not, by themselves, establish that these improvements represented intervention-specific treatment effects.

When JPE was considered as the primary outcome of the study, no consistent improvement in cervical proprioception was observed after training. At baseline, cervical flexion, extension, and sagittal-plane JPE values were higher in the CNP group than in the HC group, consistent with previous studies reporting reduced accuracy in reproducing cervical joint position among individuals with chronic neck pain [51–53]. Although cervical extension JPE decreased in the CNP group after training, the magnitude of change did not differ significantly between the groups, and no between-group difference in post-training values was found after adjustment for baseline values. For cervical flexion JPE, neither the within-group change nor the between-group difference in change was statistically significant. Nevertheless, after adjustment for baseline values and correction for multiple comparisons, post-training cervical flexion JPE remained significantly higher in the CNP group than in the HC group. These findings suggest that SIT was not followed by an overall or consistent normalization of cervical proprioception; rather, the proprioceptive difference, particularly during cervical flexion, persisted after training. Furthermore, the absence of a parallel change in objective proprioceptive performance, as measured by JPE, during the same period in which improvements in clinical symptoms were observed suggests that subjective clinical improvement and sensorimotor recovery may not occur concurrently. However, because the present study did not include a non-intervention CNP control group, either the changes or the lack of change in JPE cannot be interpreted as effects specifically attributable to SIT. Future randomized studies incorporating a non-intervention CNP control group and longer follow-up periods may help clarify the intervention-specific and time-dependent effects of SIT on cervical proprioception.

Similarly, no consistent within-group changes or baseline-adjusted between-group differences in post-training cervical range of motion were identified. Taken together, these findings suggest that altered cervical muscle activity [54,55], muscle spindle-derived afferent input [56], and the central processing of this information associated with chronic neck pain may contribute to proprioceptive differences being more resistant to change than clinical symptoms [57,58]. Although the programme included cervical stretching, deep cervical muscle activation, stabilization, and body-awareness components, the four-week intervention period and the dosage of these components may not have been sufficient to produce detectable changes in objective sensorimotor measures. However, since the current study did not compare different training durations or dosages, this explanation remains speculative.

Although some unadjusted between-group differences were observed at baseline in the neck and hand lateralization tasks, the baseline-adjusted analyses revealed no significant differences between the CNP and HC groups in post-training accuracy or reaction time. Some changes were also identified in the unadjusted within-group comparisons, including changes in neck lateralization reaction times in the HC group and left-hand accuracy in both groups. However, these results were not adjusted for multiple comparisons and should therefore be regarded as exploratory. Overall, the findings provide no evidence of a more pronounced training response in either group or of a meaningful improvement in lateralization performance attributable to SIT.

These findings are consistent with evidence suggesting that lateralization performance does not reflect a uniform deficit in chronic pain but may vary according to task characteristics, the body region assessed, and the clinical context [5,9,19,20]. Previous studies have indicated that lateralization impairments do not necessarily generalize across body regions [23,26,59,60]. In chronic neck trauma, for example, distinct reaction-time patterns have been reported despite preserved accuracy, possibly reflecting visually mediated or compensatory processing strategies [5,59]. Similarly, preserved lateralization performance despite reduced tactile acuity suggests that peripheral sensory impairment may not necessarily disrupt motor imagery [23]. Region-specific patterns reported in limb-related conditions, including knee osteoarthritis, further support the localized and context-sensitive nature of sensorimotor processing [9,19,20,26,60].

Taken together, the neck and hand lateralization findings observed in the present study suggest that CNP is not characterized by a widespread and consistent neurocognitive or motor imagery deficit and that performance should be interpreted within a task- and body-region-specific framework. In particular, the absence of baseline-adjusted between-group differences in tasks involving either a pain-free control region (the hand) or an axial region (the neck) provides no evidence that SIT produced a generalized neurocognitive effect. However, given the sample size, the absence of statistically significant differences should not be interpreted as evidence of equivalence between the groups, and the findings should be interpreted cautiously.

The persistence of impaired cervical flexion JPE in the CNP group after training, despite preserved lateralization performance, suggests that JPE and lateralization tasks may represent different dimensions of the cervical sensorimotor system. JPE relies more strongly on the processing of peripheral and cervical afferent input and the reproduction of joint position [51,61,62], whereas lateralization tasks involve central visual recognition, decision-making, and implicit motor imagery processes [24,46,63]. Therefore, the coexistence of increased cervical flexion JPE and preserved lateralization performance is not necessarily contradictory. However, because central neural mechanisms were not directly assessed in the present study, this pattern should not be interpreted as definitive evidence of compensatory cortical reorganization.

In the present study, comparisons of baseline MoCA and Stroop scores, together with the SMDs, did not indicate marked baseline differences between the CNP and HC groups. The baseline-adjusted analysis of post-training Stroop performance also revealed no significant between-group difference. Furthermore, the baseline Stroop D scores of both groups were within the normative ranges reported for individuals of a similar age, suggesting no marked impairment in selective attention or response inhibition in this sample [38]. Nevertheless, the absence of a significant between-group difference does not demonstrate that cognitive performance was definitively equivalent between the groups or exclude the possibility of cognitive impairment in the broader CNP population. Similarly, the present findings provide no evidence that SIT had a modulatory effect on general cognitive performance.

In the present study, comparisons of baseline MoCA and Stroop scores, together with the SMDs, did not indicate marked baseline differences between the CNP and HC groups. The baseline-adjusted analysis of post-training Stroop performance also revealed no significant between-group difference. Furthermore, the baseline Stroop D scores of both groups were within the normative ranges reported for individuals of a similar age, suggesting no marked impairment in selective attention or response inhibition in this sample [38]. Nevertheless, the absence of a significant between-group difference does not demonstrate that cognitive performance was definitively equivalent between the groups or that cognitive impairment is absent in the broader CNP population. Similarly, the present findings provide no evidence that SIT had a modulatory effect on general cognitive performance.

Although differences in attention, processing speed, and executive functions have been reported in chronic neck pain and other chronic pain conditions, these findings may vary depending on the assessment instrument used, the cognitive demands of the task, the clinical characteristics of pain, and the characteristics of the sample [64–68]. The Stroop test primarily assesses selective attention, cognitive flexibility, and response inhibition; it does not directly measure cervical proprioception or body-representation processes. Therefore, the absence of a comparable difference in Stroop performance despite the marked group difference in cervical flexion JPE is not necessarily contradictory. Rather, it may suggest that the cognitive processes assessed here and cervical sensorimotor processes reflect partly distinct neural mechanisms. However, given the limited range of cognitive parameters assessed and the small sample size, these findings should not be generalized as indicating that cognitive functions are entirely preserved in individuals with CNP.

### Clinical implications and limitations

The findings of this study should be interpreted in light of several limitations. First, the relatively small sample size may have limited the statistical power to detect small between-group differences and reduced the generalizability of the findings to the broader CNP population. Second, the absence of an additional CNP control group receiving either no-intervention control group or an alternative intervention makes it difficult to distinguish effects specific to SIT from the natural course of the condition, repeated assessments, participant expectations, and other nonspecific effects associated with intervention participation. When considered alongside the prospective, non-randomized, two-group pre–post quasi-experimental design, this limitation restricts causal inferences regarding intervention efficacy. Furthermore, the absence of long-term follow-up assessments precluded evaluation of whether the observed changes were sustained over time. Finally, the absence of neuroimaging or neurophysiological assessments precluded direct investigation of the central mechanisms potentially underlying the effects of motor imagery and lateralization training.

Nevertheless, the present findings suggest that changes in clinical symptoms may not necessarily be accompanied by parallel changes in objective sensorimotor performance, highlighting the importance of assessing clinical and sensorimotor outcomes together in individuals with CNP. Future large-scale randomized controlled trials incorporating no-intervention and/or active control groups and long-term follow-up assessments may allow the specific clinical effects of SIT, its effects on sensorimotor outcomes, and the persistence of these effects to be determined more reliably.

## Conclusion

Following the four-week training program, the CNP group showed statistically significant within-group reductions in pain intensity, kinesiophobia, neck-related disability, and FreNAQ-T scores, whereas no consistent improvement was observed in objective sensorimotor or cognitive measures. After adjustment for baseline values and correction for multiple comparisons, cervical flexion JPE remained higher in the CNP group than in the HC group, indicating that the proprioceptive difference in flexion persisted after training. Baseline-adjusted post-training comparisons revealed no significant between-group differences in laterality or Stroop D performance, while baseline MoCA and KVIQ scores were broadly comparable between the groups. These findings do not provide evidence of widespread impairment in cognitive performance or motor imagery ability within the study sample.. Overall, modest changes in subjective clinical outcomes were not accompanied by parallel changes in objective performance measures, highlighting the importance of assessing symptoms and sensorimotor function as complementary dimensions in CNP.

## Supporting information

S1 Appendix Structured imagery training program and exercise. (DOCX)

S2 Checklist TREND checklist for non-randomized evaluations. (PDF)

S3 Protocol Study protocol. (PDF)
