# Supplementary material for: Differential changes in cervical proprioception and motor imagery following structured imagery training in chronic neck pain
Source: PLoS One. 2026 Nov 5;21(11):e0361409. doi: 10.1371/journal.pone.0361409 (PMC13739144; doi:10.1371/journal.pone.0361409)
Supplement: Supplementary file 2 [file pone.0361409.s003.pdf]

STUDY PROTOCOL

Investigation of Lateralization and Motor Imagery Training in Individuals with Chronic Neck Pain Using Event-Related EEG Brain Oscillation Methods and Its Effect on Body Awareness: A Randomized Controlled Trial

Introduction

Chronic neck pain is one of the debilitating conditions that can impair the ability to perform daily activities, reduce productivity, and negatively affect quality of life (1). Approximately two-thirds of individuals in developed countries experience neck pain during their lifetime (1). At any given time, approximately 14–16% of the global adult population experiences neck pain, with an average lifetime prevalence of 48.5% (2). Chronic pain is defined as pain persisting for longer than three months (3).

Understanding neuroplasticity is essential for explaining the transition from acute to chronic pain (4). Local inflammation of injured tissue increases the sensitivity of peripheral sensory neurons (nociceptors), resulting in repeated abnormal afferent input to the central nervous system (5). Chronic pain is associated with neuroplastic changes in the brain, leading to abnormal cortical reorganization and the formation of abnormal neural circuits within subcortical structures. As a consequence of chronic pain, disturbances in body schema perception, reduced ability to perceive somatosensory differences, impaired movement quality, and difficulties in performing complex movements may occur (6). Altered cervical afferent input to higher centers may consequently impair cervical proprioception (7).

One of the major problems in individuals with neck pain is that impaired cervical proprioception may lead to deficits in cervical sensorimotor control. Previous studies have emphasized that interventions should primarily focus on specific programs targeting different aspects of sensorimotor control impairments, particularly those aimed at improving proprioception and muscle coordination (8–10).

Rationale

Motor imagery (MI) is an intervention developed particularly to reduce pain and improve function in individuals with chronic pain conditions. It is well established that imagined movements and actual movements share similar neural pathways and activation patterns (11). During motor imagery, brain regions activated during actual movement, particularly motor cortical areas, are similarly activated. This approach enables activation of the premotor cortex without direct activation of the primary motor cortex. Consequently, movement-related neural signals are generated without actual execution of movement (12,13).

Laterality Training (LT) is a form of implicit motor imagery that requires individuals to identify whether an image of a body part belongs to the left or right side as accurately and rapidly as possible (14,15). Differences in LT performance have been suggested to reflect alterations in central nervous system functioning, judgment errors, and changes in body representations (16). LT is considered a complex cognitive task involving cognitive, sensory, motor, and behavioral processes and has been associated with these functions (16). Previous research has demonstrated a strong correlation between laterality performance and proprioception (17).

Individuals with chronic pain have been shown to exhibit reduced ability to perform left–right judgments (limb laterality recognition). The ability to distinguish left from right is closely related to one's capacity to position the body in space. Laterality training aims to retrain higher cortical functions of the right cerebral hemisphere, including global visual processing, visual skills, and body schema perception, thereby improving these abilities (18,19).

In some individuals with chronic pain, merely thinking about moving a painful body part may lead to excessive activation of motor-related cortical regions, including the supplementary motor area (SMA), cingulate motor area, and primary motor cortex (M1). This heightened activation may exacerbate pain-related cognitive processes and reinforce painful experiences (20).

OBJECTIVES

For these reasons, investigating cognitive involvement and cortical activity changes in individuals with chronic neck pain has emerged as an important area of research. Electroencephalography (EEG), a non-invasive technique, is one of the methods used to measure cortical activity (21).

Primary Objective

In light of this background, the primary objective of the present study is to investigate whether brain activity changes at the cortical level during laterality and motor imagery training by measuring the electrical activity of the brain using EEG.

Secondary Objectives

The secondary objectives are:

1. To examine the relationship between EEG activity and kinesiophobia, neck pain intensity, and proprioception in individuals with chronic neck pain.
2. To evaluate the relationships among kinesiophobia, neck pain intensity, and proprioception in individuals with chronic neck pain.

To the best of our knowledge, no previous study has investigated the relationships among pain, proprioception, kinesiophobia, laterality training, and EEG brain activity in individuals with chronic neck pain. Therefore, this study aims to provide novel findings regarding these associations.

Hypotheses

Hypothesis 1: EEG activity will demonstrate cortical-level alterations in individuals with chronic neck pain.

Hypothesis 2: EEG activity will be associated with kinesiophobia, neck pain intensity, two-point discrimination performance, and proprioception in individuals with chronic neck pain.

Hypothesis 3: Kinesiophobia, neck pain intensity, two-point discrimination performance, and proprioception will be significantly associated with one another in individuals with chronic neck pain.

STUDY DESIGN

Individuals with chronic neck pain who meet the inclusion and exclusion criteria will be recruited from the FSM Medical Center by Dr. Emel Deniz Şahin. On Day 1, all baseline assessments and outcome measures will be conducted by a physiotherapist. On Day 2,

participants will be referred to the EEG Laboratory of Istanbul Medipol University for EEG recordings. Transportation expenses will be covered by the physiotherapist.

The study will be conducted in four phases:

Phase 1

Participants' demographic characteristics will be recorded. The following assessments will be performed:

- Neuropsychological tests (NPT)
- Pain assessment
- Kinesiophobia assessment
- Neck Disability Index
- Motor imagery assessment
- Two-point discrimination test
- Muscle activation assessment
- Proprioceptive sensory tests

Phase 2

Participants will undergo resting-state EEG recording, followed by EEG recordings during the Oddball Paradigm and the Laterality Imagery Paradigm.

Phase 3

Participants will receive a 3-week training program. During the first two weeks, laterality training will be administered daily. Beginning in the second week, motor imagery training will be added to the laterality training program and continued throughout the third week. Motor imagery training will be provided twice weekly, for a total of six sessions.

Participants will receive a total of 20 treatment sessions, consisting of 14 laterality training sessions and 6 motor imagery training sessions.

Following completion of the training program, participants will be reassessed using:

- Pain assessment
- Kinesiophobia assessment
- Neck Disability Index
- Montreal Cognitive Assessment (MoCA)
- Two-point discrimination test
- Muscle activation assessment
- Proprioceptive sensory tests

Phase 4

After completion of the three-week laterality and motor imagery training program and all post-intervention assessments, participants will undergo a second EEG evaluation, including resting-state EEG recording, the Oddball Paradigm, and the Laterality Imagery Paradigm.

Control Group

Participants who meet the eligibility criteria for the control group will be recruited accordingly. Transportation expenses will be covered by the physiotherapist.

The control group will also undergo four phases:

Phase 1

Participants' demographic characteristics will be recorded. The following assessments will be performed:

- Neuropsychological tests (NPT)
- Pain assessment
- Kinesiophobia assessment
- Neck Disability Index
- Motor imagery assessment
- Two-point discrimination test
- Muscle activation assessment
- Proprioceptive sensory tests

Phase 2

Participants will undergo resting-state EEG recording, followed by EEG recordings during the Oddball Paradigm and the Laterality Imagery Paradigm.

Phase 3

Participants will receive the same 3-week training program. During the first two weeks, laterality training will be administered daily. Beginning in the second week, motor imagery training will be added and continued throughout the third week. Motor imagery training will be provided twice weekly, for a total of six sessions.

Participants will receive a total of 20 treatment sessions, consisting of 14 laterality training sessions and 6 motor imagery training sessions.

Following completion of the training program, participants will be reassessed using:

- Pain assessment
- Kinesiophobia assessment
- Neck Disability Index
- Montreal Cognitive Assessment (MoCA)
- Two-point discrimination test
- Muscle activation assessment
- Proprioceptive sensory tests

Phase 4

Following completion of the training program and all post-intervention assessments, participants will undergo a second EEG evaluation, including resting-state EEG recording, the Oddball Paradigm, and the Laterality Imagery Paradigm.

Graded Motor Imagery Training Program

Graded Motor Imagery (GMI) is a rehabilitation approach that focuses on the gradual training of brain function. It is a therapeutic strategy based on the biopsychosocial model and is designed to address pain, activity limitations, and functional impairments. By progressively exposing the brain to visual information, GMI aims to facilitate the gradual reorganization of cortical activity and reduce abnormal cortical disinhibition (15).

GMI consists of three components based on the processes involved in movement generation (movement intention, motor planning and preparation, movement initiation, and execution), as well as the concepts of the neuromatrix, pain science, and fear of movement. These components include:

1. **Laterality Training** (left–right discrimination training)
2. **Motor Imagery** (mental rehearsal of movement)
3. **Mirror Therapy** (visual feedback provided through a mirror)

Laterality training (implicit motor imagery) involves identifying whether a limb belongs to the left or right side of the body or, in the case of the spine, determining whether the individual is rotated to the left or right. Motor imagery (explicit motor imagery) involves mentally rehearsing movements and postures using internal, external, and kinesthetic imagery strategies.

The sequential application of these three stages is intended to progressively activate cortical motor networks and promote cortical reorganization (15,39,40).

Details regarding the stages and progression of the Graded Motor Imagery training program are presented below.

Stages	Starting Point	Progression	Considerations
**Stage 1: Laterality Training**	Twenty simple laterality cards/images are used. The number of correct responses and response time are recorded.	The speed, number, and difficulty of the images are gradually increased until laterality performance improves.	Does the participant experience pain while viewing the images? If so, easier images that do not provoke pain are selected and used.
**Stage 2: Motor Imagery**	The participant is instructed to imagine the affected body part in different positions.	The participant is instructed to imagine the affected body part moving through different positions and movements.	Does the participant experience pain during imagery? If so, the program returns to laterality training and non-pain-provoking images are used.

Left/Right Neck Laterality Training

Recognise™ is an application designed for use on both iPhone and iPad devices. As part of the Graded Motor Imagery (GMI) rehabilitation program, it assesses the accuracy of left/right discrimination judgments (41).

Wallwork et al. (42) investigated the feasibility of left/right neck rotation judgment tasks in individuals with neck pain using the Recognise™ application (NOI Group, Adelaide, Australia) and reported that the method was appropriate and feasible for this population. Accordingly, laterality training in the present study will be conducted using images obtained from the Recognise™ application (NOI Group, Adelaide, Australia).

Participants will view images presented on a computer screen and will be asked to determine whether the model's head is rotated to the left or right relative to the shoulders. Participants will be instructed to respond as quickly and accurately as possible without guessing (42).

A Laterality Imagery Paradigm will be developed based on these procedures and will consist of 40 images in total, including 20 right-rotation and 20 left-rotation neck images.

Motor Imagery Training

Participants will receive motor imagery training involving the mental rehearsal of head movements performed in specific positions and at predetermined angles.

EEG Recording Procedure

EEG recordings will be performed in the following sequence:

1. Resting-state EEG recording consisting of 4 minutes with eyes open and 4 minutes with eyes closed.
2. EEG recording during the Visual Oddball Paradigm following the resting-state EEG assessment.
3. EEG recording during the Laterality Training Paradigm following completion of the Oddball Paradigm.

Instructions for the Laterality Imagery Paradigm

Before the initiation of the Laterality Imagery Paradigm, participants will receive the following instructions:

- When an image appears on the screen, they should determine whether the depicted body position corresponds to a left or right neck rotation.
- If the image represents a right-sided neck rotation, they should press the right-arrow key on the keyboard.
- If the image represents a left-sided neck rotation, they should press the left-arrow key on the keyboard.
- When a fixation cross (+) appears on a black screen, they should remain relaxed and avoid engaging in any specific thoughts or mental imagery.

Laterality Training Paradigm

For each trial, the sequence will be as follows:

4 s Picture → 3–5 s Interstimulus Interval (ISI) → 1 s Fixation Cross (+)

The average duration of each trial will be approximately 10 seconds.

The paradigm will consist of a total of 40 images, resulting in an overall duration of approximately 400 seconds (6–7 minutes).

At the beginning of each trial, an image depicting a neck position will be presented on the screen for 4 seconds. This will be followed by a black screen with an interstimulus interval (ISI) of 3–5 seconds. Subsequently, a fixation cross (+) will be displayed for 1 second. The next image will then appear, and this sequence will continue until all 40 images have been presented.

The paradigm will include 20 images representing right neck rotation and 20 images representing left neck rotation. Participants will be instructed to identify the direction of neck rotation as quickly and accurately as possible. If the image depicts right neck rotation, participants will press the right-arrow key on the keyboard; if the image depicts left neck rotation, they will press the left-arrow key.

Visual Oddball Paradigm

Event-Related Potentials (ERPs) are electrical brain responses elicited by specific stimuli during EEG recordings (43). To obtain ERPs, markers are synchronized with stimulus presentation during continuous EEG recording, and stimuli are presented with variable interstimulus intervals. ERP paradigms are widely used to investigate cognitive processes such as attention, concentration, memory, and decision-making (44,45).

The validity and usefulness of the Oddball Paradigm have been demonstrated in previous studies (46–48). Stevens et al. (46) compared auditory and visual oddball paradigms using event-related functional magnetic resonance imaging (fMRI) and reported that visual stimuli activated a greater extent of cortical regions than auditory stimuli. Therefore, a visual oddball paradigm was selected in the present study to complement the motor imagery training intervention.

During the task, participants will be presented with a randomized sequence of visual stimuli consisting of different colors. Participants will be instructed to pay attention to the designated target stimulus and to discriminate between target and non-target stimuli. They will be informed that no response is required when non-target stimuli appear.

Participants will be instructed to press the “0” key whenever a target stimulus is presented and to withhold responses to standard stimuli. Reaction time and target-detection accuracy will be recorded for each participant.

The outcomes obtained from the visual oddball paradigm will be used to evaluate cognitive processes such as attention, learning, memory, and information processing and to investigate how these processes may be influenced by motor imagery training.

Oddball Paradigm Stimuli

The visual oddball paradigm will consist of a total of 200 stimuli, including 120 non-target stimuli, 40 target stimuli, and 40 distractor stimuli.

Each stimulus will be presented for 1 second. The interstimulus interval (ISI) will vary randomly between 2 and 4 seconds.

The stimuli will be presented using the following color scheme:

- **Non-target stimuli:** dark gray
- **Target stimuli:** bright white
- **Distractor stimuli:** light gray

Participants will be instructed to respond only to target stimuli while ignoring both non-target and distractor stimuli.

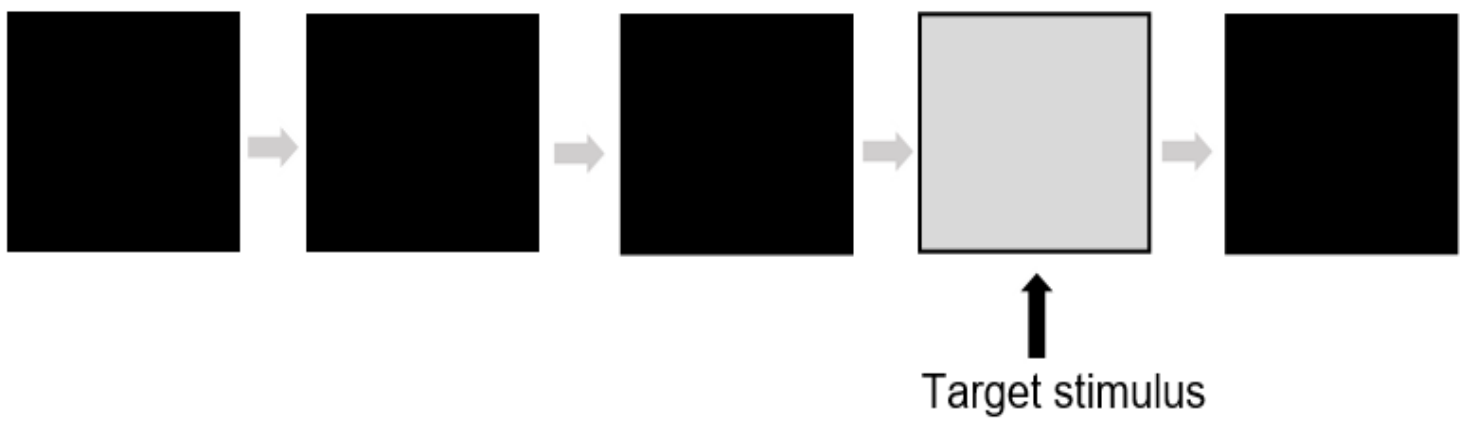

Participant Selection

Inclusion Criteria for the Chronic Neck Pain Group

Participants will be eligible for inclusion if they meet the following criteria:

- Aged between 18 and 65 years.
- Presence of chronic neck pain lasting longer than 3 months.
- Adequate understanding of the Turkish language.
- Ability to use a smartphone and/or computer.
- Literate and able to understand digital media content.
- A score of 21 or higher on the Montreal Cognitive Assessment (MoCA).
- A pain intensity score of $\geq$70 mm on the Visual Analog Scale (VAS).
- Grade II or III according to the Neck Pain Task Force Classification.

Exclusion Criteria

Participants will be excluded if they:

- Have received treatment for neck pain within the previous 6 months.
- Have undergone cervical spine surgery.
- Have suspected malignant or systemic disease.

- Have a neurological disorder or injury.
- Report vertigo, nausea, or visual disturbances.

Inclusion Criteria for the Healthy Control Group

Participants will be eligible for the healthy control group if they:

- Do not experience neck or back pain that interferes with their normal daily activities.
- Have never received medical treatment or intervention for a neck-related condition.

Outcome Assessments

Following the provision of written informed consent, all participants will undergo the following assessments.

Assessments for Participants with Chronic Neck Pain

- Demographic Information Form
- Neuropsychological Tests (NPT)
- Motor Imagery Assessment
- Neck Pain Task Force Classification
- Pain Intensity Assessment
- Kinesiophobia Assessment
- Neck Disability Assessment
- Two-Point Discrimination Test (TPD)
- Proprioception Assessment
- Treatment Adherence Assessment

Assessments for Healthy Controls

- Demographic Information Form
- Neuropsychological Tests (NPT)
- Motor Imagery Assessment

Assessments

Following the provision of written informed consent, all participants will undergo the following assessments.

Cognitive Function Assessment

Neuropsychological Tests

The neuropsychological assessment battery will include the Montreal Cognitive Assessment (MoCA), the Benton Visual Retention Test, and the Stroop Test.

Montreal Cognitive Assessment (MoCA)

The Montreal Cognitive Assessment (MoCA) will be used to evaluate participants' cognitive status and to determine eligibility for study participation. The MoCA assesses seven cognitive

domains, including attention, concentration, memory, language, calculation, visuospatial abilities, reasoning, and orientation. Administration requires approximately 10 minutes. The maximum possible score is 30, and scores of 21 or above will be considered indicative of normal cognitive functioning. The Turkish version of the MoCA has been validated by Özdilek and Kenangil (22,23).

Benton Visual Retention Test

The Benton Visual Retention Test (BVRT) will be used to assess visual memory, visual perception, and visuoconstructive abilities (24). The test consists of two forms: a drawing (reproduction) form and a recognition form. The recognition form comprises 15 stimulus cards and 15 multiple-choice response cards. Each stimulus card is presented for 10 seconds, after which participants are asked to identify the previously displayed figure from a set of alternatives. Administration of all 15 items requires approximately 5–10 minutes. Test performance provides an assessment of visual memory function.

Stroop Test

The Stroop Test is a three-part cognitive control task originally developed by J.R. Stroop in 1935 (25). The Stroop effect occurs when participants are required to name the color of a word while the word itself denotes a different color. This interference effect has been widely accepted as a gold-standard measure of attentional control (26). In the first section, participants read color names as quickly as possible. In the second section, they identify the colors of colored dots. In the third section, they name the ink color of incongruent color words as quickly and accurately as possible. The Turkish validity and reliability study was conducted by Karakaş et al. (27).

Motor Imagery Assessment

Motor imagery ability will be assessed using the Movement Imagery Questionnaire-3 (MIQ-3). The Turkish validity and reliability study was conducted by Dilek et al. (28). The questionnaire consists of 12 items and evaluates three domains: external visual imagery, internal visual imagery, and kinesthetic imagery. For each task, participants first physically perform the movement and subsequently imagine the movement according to the specified imagery modality. Responses are scored on a 7-point scale ranging from 1 (“very difficult to see/feel”) to 7 (“very easy to see/feel”). Scores for each subscale are calculated separately by averaging the corresponding four items (28).

Neck Pain Task Force Classification

The Neck Pain Task Force Classification is a four-grade classification system designed to facilitate the interpretation of scientific evidence regarding neck pain severity (29).

- **Grade I:** No signs or symptoms suggestive of major structural pathology, with little or no interference in daily activities. Neck pain, stiffness, and tenderness may be present.
- **Grade II:** No signs or symptoms of major structural pathology; however, neck pain substantially interferes with daily activities and may require early intervention.
- **Grade III:** No major structural pathology is present, but neurological signs such as reduced deep tendon reflexes, muscle weakness, and/or sensory deficits are observed.

- **Grade IV:** Signs or symptoms suggestive of major structural pathology, such as fracture, myelopathy, neoplasm, or systemic disease, requiring urgent investigation and treatment.

Pain Intensity Assessment

Pain intensity will be assessed using the Visual Analog Scale (VAS). The VAS consists of a 10-cm horizontal line, where 0 represents "no pain" and 10 represents "the worst pain imaginable." Participants will be asked to indicate their current pain intensity by marking the appropriate point on the scale (30).

Kinesiophobia Assessment

Fear of movement will be evaluated using the Tampa Scale for Kinesiophobia (TSK). The TSK is a 17-item questionnaire developed to assess fear of movement and reinjury in individuals with musculoskeletal pain. Each item is scored on a 4-point Likert scale ranging from 1 ("strongly disagree") to 4 ("strongly agree"). Items 4, 8, 12, and 16 are reverse scored. Total scores range from 17 to 68, with higher scores indicating greater levels of kinesiophobia. The Turkish version has demonstrated satisfactory reliability and validity (31).

Neck Disability Assessment

Neck-related disability will be evaluated using the Neck Disability Index (NDI). The Turkish version of the NDI has been validated by Telci et al. (32). The questionnaire consists of 10 sections assessing pain intensity, personal care, lifting, reading, headaches, concentration, work, driving, sleeping, and recreational activities. Each section is scored from 0 to 5, yielding a total score ranging from 0 to 50, where higher scores indicate greater disability. To account for unanswered items, scores will be converted into percentages. Disability levels of 8% or lower will be considered indicative of no disability (32).

Two-Point Discrimination Test (TPD)

Tactile acuity in the cervical region will be assessed using the Two-Point Discrimination (TPD) test, which has demonstrated acceptable validity and reliability in previous studies (33). Measurements will be performed using a Vernier caliper. This procedure is entirely non-invasive and involves only light contact of the instrument with the skin surface without compromising tissue integrity.

Two-point discrimination assesses the innervation density of slowly adapting sensory receptors. Greater distances required to distinguish two points indicate reduced cutaneous sensory acuity. Test results will be interpreted as follows: 0–5 mm = normal sensation; 6–10 mm = diminished sensation; 11–15 mm = poor sensation; perception of only one point = protective sensation; inability to perceive any point = anesthesia (34).

Proprioception Assessment

Cervical joint position error will be assessed using a Dualer IQ Digital Inclinometer (J-Tech Medical, Midvale, UT, USA). Digital inclinometry has been widely used in spinal assessment protocols and has been endorsed by the American Medical Association (35).

The device enables clinicians to evaluate both range of motion and proprioceptive function using dynamic inclinometry procedures similar to those employed in conventional goniometric assessments (36). Previous studies have demonstrated acceptable test–retest reliability of digital inclinometers for the assessment of spinal range of motion (35,37).

Study Timeline

The participant recruitment period is planned to extend over 15 months. Recruitment is scheduled to commence on July 1, 2023, and to be completed by October 1, 2024.

Statistics

Sample Size Calculation

Individuals with chronic neck pain and healthy control participants will be recruited on a voluntary basis according to the relevant eligibility criteria. Participants will be included in either the chronic neck pain group or the healthy control group based on their clinical status; no random allocation will be performed. An a priori sample size calculation was performed using G*Power software (version 3.1.9.2) for the group × time interaction in a two-group, two-time-point repeated-measures ANOVA. The calculation assumed a medium interaction effect size (Cohen's $f = 0.25$), an alpha level of 0.05, 80% statistical power, a correlation of 0.50 among repeated measurements, and a nonsphericity correction of 1.00. These parameters yielded a required total sample size of 34 participants, corresponding to 17 participants per group, with an actual power of 0.807.

Statistical Analysis

Statistical analyses will be performed using SPSS software (version 21.0; SPSS Inc., Chicago, IL, USA). Following assessment of data distribution, both parametric and non-parametric analyses will be conducted as appropriate.

Descriptive statistics for parametric variables will be presented as mean ± standard deviation, whereas non-parametric variables will be reported as frequencies, percentages, and quartile values.

Parametric variables will be analyzed using the Student's t-test, while non-parametric variables will be compared using the Mann–Whitney U test.

Changes over time and between-group differences involving both the study and control groups will be analyzed using Repeated Measures Analysis of Variance (Repeated Measures ANOVA).

A p-value of <0.05 will be considered statistically significant for all analyses.

Results

The collected data will be analyzed using the appropriate statistical methods described above.

Discussion

The findings obtained from the statistical analyses will be interpreted and discussed in the context of the existing literature.

References

1. Carvalho, R.Cd., Maglioni, C.B., Machado, G.B., Araújo, J.Ed., Silva, J.R.Td., Silva, M.Ld. 2018. "Prevalence and characteristics of chronic pain in Brazil: a national internet-based survey study", BrJP, 1(4), 331-338.

2. Genebra, C.V.D.S., Maciel, N.M., Bento, T.P.F., Simeão, S.F.A.P., Vitta Ad. 2017. “Prevalence and factors associated with neck pain: a population-based study”, Brazilian journal of physical therapy, 21(4), 274-80.

3. Hague, M., Shenker N. 2014. “How to investigate: Chronic pain”, Best Practice & Research Clinical Rheumatology”, 28(6), 860-74.

4. Brumagne, S., Diers, M., Danneels, L., Moseley, G.L., Hodges P.W. 2019. “Neuroplasticity of sensorimotor control in low back pain”, Journal of orthopaedic & sports physical therapy, 49(6), 402-14.

5. Yam, M.F., Loh, Y.C., Tan, C.S., Khadijah Adam, S., Abdul Manan, N., Basir R. 2018. “General pathways of pain sensation and the major neurotransmitters involved in pain regulation”, International journal of molecular sciences, 19(8), 2164.

6. Schwoebel, J., Coslett, H.B., Bradt, J., Friedman, R., Dileo, C. 2002. “Pain and the body schema: effects of pain severity on mental representations of movement”, Neurology, 59(5), 775-7.

7. Reddy, R.S., Tedla, J.S., Dixit, S., Abohashrh, M. 2019. “Cervical proprioception and its relationship with neck pain intensity in subjects with cervical spondylosis”, BMC musculoskeletal disorders, 20(1), 1-7.

8.Treleaven, J. 2008. “Sensorimotor disturbances in neck disorders affecting postural stability, head and eye movement control”, Man Ther, 13(1), 2-11.

9.Treleaven J. 2008. “Sensorimotor disturbances in neck disorders affecting postural stability, head and eye movement control–Part 2: case studies”, Man Ther, 13(3), 266-275.

10. Peng, B., Yang, L., Li, Y., Liu, T., & Liu, Y. 2021. “Cervical Proprioception Impairment in Neck Pain Pathophysiology, Clinical Evaluation, and Management: A Narrative Review”, Pain and Therapy, 10(1), 143.

11. Jeannerod, M. 1994. “The representing brain: Neural correlates of motor intention and imagery”, Cambridge University Press, 187-202.

12. Hétu, S., Grégoire, M., Saimpont, A., Coll, M.P., Eugène, F., Michon, P.E., et. al. 2013. “ The neural network of motor imagery: an ALE meta-analysis”, Neurosci Biobehav Rev, (37), 930-949.

13. Solodkin, A., Hlustik, P., Chen, E.E., Small, S.L. 2004. “Fine modulation in network activation during motor execution and motor imagery”, Cereb Cortex, 14(11), 1246-1255.

14. Osuagwu, B.A., Vuckovic, A. 2014. “Similarities between explicit and implicit motor imagery in mental rotation of hands: An EEG study”, Neuropsychologia, 65(0), 197-210.

15. Moseley, G.L. 2012. “The Graded Motor Imagery Handbook”, Noigroup publications, Australia.

16. Pelletier, R., Bourbonnais, D., Higgins, J., Mireault, M., Danino, M.A., & Harris, P.G. 2018. “Left Right Judgement Task and Sensory, Motor, and Cognitive Assessment in Participants with Wrist/Hand Pain”, Rehabilitation Research and Practice, 2018, 1-13.

17. Elsig, S., Luomajoki, H., Sattelmayer, M., Taeymans, J., Tal-Akabi, A., & Hilfiker, R. 2014. “Sensorimotor tests, such as movement control and laterality judgment accuracy, in persons with recurrent neck pain and controls. A case-control study”, Manual Therapy, 19(6), 555-561.

18. Breckenridge, J., Ginn, K., Wallwork, S., McAuley, J. 2016. “ Does Upper Limb Pain Affect the Left/Right Judgment Task? A Systematic Review and Meta-analysis”, HAND, 2016(11), 136.

19. Adın, R.M., Kuru Ç.A. 2018. “Aşamalı Motor İmgeleme Yaklaşımı/Graded Motor Imagery Approach”, In book: Fizyoterapi Seminerleri E Kitap, 17-23.

20. Osumi, M., Sumitani, M., Nishi, Y., Nobusako, S., Dilek, B., Morioka, S. 2021. “Fear of movement-related pain disturbs cortical preparatory activity after becoming aware of motor intention”, Behavioural brain research, 411, 113379.

21. Wolpaw, J.R., Loeb, G.E., Allison, B.Z., Donchin, E., Nascimento, O.Fd, Heetderks, W.J., Nijboer, F., Shain, W.G., Turner, J.N. 2006. “BCI meeting 2005-workshop on signals and recording methods”, IEEE Transactions on Neural Systems and Rehabilitation Engineering, 14(2), 138-141.

22. Özdilek B. ve Kenangil, G. 2014. “Validation of the Turkish Version of the Montreal Cognitive Assessment Scale (MoCA-TR) in patients with Parkinson’s disease”, The Clinical Neuropsychologist, 28(2), 333-343.

23. Nasreddine, Z.S., Phillips, N.A., Bédirian, V., Charbonneau, S., Whitehead, V., Collin, I., Chertkow, H. 2005. “The Montreal Cognitive Assessment, MoCA: a brief screening tool for mild cognitive impairment”, Journal of the American Geriatrics Society, 53(4), 695-699.

24. Manna, C.G., et al. 2011. Sayfa 392-304. “Benton Visual Retention Test in Encyclopedia of Clinical Neuropsychology”, Editors: Kreutzer, J.S., DeLuca, J., Caplan, B. New York: Springer.

25. Stroop, J.R. 1935. “Studies of interference in serial verbal reactions”, Journal of experimental psychology, 18, 643.

26. MacLeod, C.M. 1992. “The Stroop task: The" gold standard" of attentional measures”, Journal of Experimental Psychology: General, 121(1), 12.

27. Karakaş, S., Irak, M., Ersezgin, Ö. 1998. “Wisconsin Kart Eşleme Testi (WCST) ve Stroop Testi TBAG formu puanlarının test içi ve testler-arası ilişkileri”, X Ulusal Psikoloji Kongresi özet kitabı, 44.

28. Dilek, B., Ayhan, C., Yakut, Y. 2020. “Reliability and validity of the Turkish version of the movement imagery questionnaire-3: Its cultural adaptation and psychometric properties”, Neurol Sci Neurophysiol, 37, 221-227.

29. Haldeman, S., Carroll, L., Cassidy, J. D., Schubert, J., & Nygren, Å. 2008. “The Bone and Joint Decade 2000–2010 Task Force on Neck Pain and Its Associated Disorders”, European Spine Journal, 17(1), 5-7.

30. Downie, W. W., Leatham, P. A., Rhind, V. M., Wright, V., Branco, J. A., & Anderson, J. A. 1978. “Studies with pain rating scales”, Annals of the Rheumatic Diseases, 37(4), 378-381.

31. Yılmaz, Ö.T., Yakut, Y., Uygur, F., Uluğ, N. 2011. “Tampa kinezyofobi ölçeği‟nin Türkçe versiyonu ve test-tekrar test güvenirliği”, Fizyoterapi Rehabilitasyon, 22(1), 44-49.

32. Telci, E.A., Karaduman, A., Yakut, Y., Aras, B., Simsek, B.E., Yagli, N. 2009. “The cultural adaptation, reliability, and validity of neck disability index in patients with neck pain: A turkish version study”, Spine, 34(16), 1732-1735.

33. Harvie, D.S., Kelly, J., Buckman, H., Chan, J., Sutherland, G., Catley, M., et al. 2017. “Tactile acuity testing at the neck: A comparison of methods”, Musculoskelet Sci Pract, 32, 23-30.

34. Öksüz, Ç., Akel, B.S. 2016. Sayfa 299-312. Fizyoterapi ve Rehabilitasyon Cilt 1. Editör: Karaduman A.A., Yılmaz Ö.T. Ankara: Hipokrat Kitabevi ve Pelikan Kitabevi.

35. Nitschke, J.E., Nattrass, C.L., Disler, P.B., Chou, M. J., Ooi, K.T. 1999. “Reliability of the American Medical Association guides' model for measuring spinal range of motion. Its implication for whole-person impairment rating”, Spine, 24(3), 262-268.

36. Tousignant, M., Boucher, N., Bourbonnais, J., Gravelle, T., Quesnel, M., Brosseau, L. 2001. “Intratester and intertester reliability of the Cybex electronic digital inclinometer (EDI-320) for measurement of active neck flexion and extension in healthy subjects”, Manual therapy, 6(4), 235-241.

37. Santos, C.M.D., Ferreira, G., Malacco, P.L., Sabino, G.S., Moraes, G.F.D.S., Felício, D.C. 2012. “Intra and inter examiner reliability and measurement error of goniometer and digital inclinometer use”, Revista Brasileira de Medicina do Esporte, 18, 38-41.

38. Cadmus-Bertram, L., Irwin, M., Alfano, C., Campbell, K., Duggan, C., Foster-Schubert, K., et al. 2014. “Predicting adherence of adults to a 12-month exercise intervention”, J Phys Act Health, 11(7), 1304-12.

39. Moseley, G.L. 2006. “Graded motor imagery for pathologic pain: a randomized controlled Trial”, Neurology, 67(12), 2129-34.

40. Priganc, V.W., Stralka, S.W. 2011. “Graded motor imagery”, J Hand Ther, 24(2),164-9.

41. Moseley, G.L. 2004. “Why do people with complex regional pain syndrome take longer 397 to recognize their affected hand?”, Neurology, 62, 2182-6.

42. Wallwork, S.B., Butler, D.S., Fulton, I., Stewart, H., Darmawan, I., Moseley, G.L. 2013. “Left/right neck rotation judgments are affected by age, gender, handedness and image rotation”, Manual Therapy, 18(3), 225-230.

43. Sur, S., Sinha, V.K. 2009. “Event-related potential: An overview”, Ind Psychiatry J, 18(1), 70-73.

44. Amin, H.U., Malik, A.S., Kamel, N., Chooi, W.T., Hussain, M. 2015. “P300 correlates with learning & memory abilities and fluid intelligence”, J NeuroEng Rehabil, 12(1), 87.

45. Huettel, S.A., McCarthy, G. 2004. “What is odd in the oddball task? Prefrontal cortex is activated by dynamic changes in response strategy”, Neuropsychologia, 42(3), 379–386.

46. Stevens, A.A., Skudlarski, P., Gatenby, J.C., Gore, J.C. 2000. “Event-related fMRI of auditory and visual oddball tasks”, Magn Reson Imaging, 18(5), 495-502.

47. Fukami, T., Shimada, T., Akatsuka, T., Ishikawa, F., Ishikawa B., Saito Y. 2003. "The temporal change of activations in the visual target oddball stimulus", IEEE EMBS Asian-Pacific Conference on Biomedical Engineering, 158-159.

48. Fajkus, J., Mikl, M., Shaw, D.J., Brázdil, M. 2015. "An fMRI investigation into the effect of preceding stimuli during visual oddball tasks", J. Neurosci. Methods, 251, 56-61.
