# Supplementary material for: Differential changes in cervical proprioception and motor imagery following structured imagery training in chronic neck pain
Source: PLoS One. 2026 Nov 5;21(11):e0361409. doi: 10.1371/journal.pone.0361409 (PMC13739144; doi:10.1371/journal.pone.0361409)
Supplement: Supplementary file 3 [file pone.0361409.s001.docx]

**Supporting information**

**Appendix A**

**Description of Lateralization and motor imagery training**

The study included a 4-week structured imagery training program conducted via videoconference. During the first two weeks, lateralization imagery training was administered daily, followed by motor imagery training three times per week in the final two weeks. A total of 20 treatment sessions were delivered: 14 sessions of lateralization and 6 sessions of motor imagery.

1. **Lateralization training**

Fourteen sessions of lateralization training were applied 7 days a week for 2 weeks. At this stage, 20 images obtained from the “Recognise™ Neck and Hand” application used in the lateralization assessment (<https://www.noigroup.com/>) were used (Moseley et al, 2012; Wajon, 2014). Participants were encouraged to continue using the application outside of supervised sessions. To this end, the “Recognise™ Neck and Hand” application was downloaded to the participants' phones, and they were asked to perform the right/left discrimination of neck and hand images every day a week, both during supervised sessions and at home, without focusing on their own neck and hands.

2. **Motor imagery trainig**

Six sessions of motor imagery trainig were applied 3 days a week for last 2 weeks. Each session lasted 30 minutes under the supervision of a physiotherapist via video conferencing. The intervention program consisted of a combination of body awareness training and neck exercises.

**2.1 Body Awareness Exercises**

**2.1.1 Positioning**

- The patient was seated upright on a chair.
- The feet were bare and placed flat on the floor with full contact.
- The hands were resting on the knees.
- The patient was instructed to relax the shoulders.
- The eyes were closed.

**2.1.2 Body Awareness Instructions**

- The patient was asked to feel the full contact of the feet with the floor.
- Starting from the little toe, the patient was instructed to focus on each toe and feel the big toe.
- Attention was directed toward the knees, sensing both the right and left kneecaps.
- The patient was instructed to notice the contact between the hands and the thighs.
- Attention was directed to the hips and the contact between the hips and the chair.
- The patient was asked to feel the hips spreading and settling into the chair.
- The contact between the back and the backrest of the chair was acknowledged.
- Attention was moved from the hands toward the elbows, sensing both elbows.
- The patient was instructed to focus on the shoulders, release them, and feel them relax.
- Finally, attention was directed to the neck muscles, sensing relaxation in both the right and left sides of the neck.

**2.2 Motor imagery training**

The program were performed by instructing the patient to mentally simulate movements of the relevant body part without executing any physical movement. The exercises were conducted in a relaxed seated position with the eyes closed. The patient was asked to visualize the relevant body part and imagine the movement from a first-person (internal) perspective. The movement was mentally simulated in a slow, controlled, and pain-free manner, while imagining the associated muscle activation and joint motion. The exercises were performed separately for the right and left sides. Each movement was repeated 5–10 times, and each imagery trial lasted approximately 5–10 seconds.

Before initiating the imagery exercises, the movements and the use of the theraband were demonstrated once to the patient. The patient was instructed to feel the theraband and perceive its resistance.

Exercises should not cause much pain in participant and should not cause an increase in neck pain during the day after exercise.

**2.2.1 Motor imagery–based stretching**

Purpose: stretching the neck

Neck exercises consisted of active stretching movements performed with concurrent motor imagery. The patient was instructed to place the hand on top of the head and imagine bringing the right ear toward the right shoulder, holding the stretch for 5 seconds at the point of tension. The same procedure was then performed for the left side by imagining bringing the left ear toward the left shoulder. The patient then returned to the starting position, placed both hands under the chin, and was instructed to imagine moving the head backward in a controlled manner, stopping at the pain threshold without forcing the movement. Finally, with the hand placed on the midline of the head, the patient was instructed to imagine bringing the chin toward the chest. Each position was maintained for 5 seconds, and all exercises were performed for 5 repetitions.

**2.2.2 Motor imagery–based chin-tuck stretching**

Purpose: stretching the back of the neck

Participants, in a sitting position with the head straight and facing forward, gently pulled their jaws back towards their neck and flexed their heads slightly. Maintaining this position for 5 seconds, they returned to the starting position and completed the movement. They imagined and repeated this movement 5 times.

**2.2.3 Motor imagery–based activation of deep neck muscles**

Purpose: To activate and strengthen the deep neck muscles

Isometric activation exercises were performed as resisted contractions targeting the cervical musculature. The patient was instructed to place the right hand on the lateral aspect of the head and imagine applying pressure by pushing the head against the hand, maintaining resistance for 5 seconds. The same procedure was then performed on the left side. Next, the hand was placed on the forehead, and the patient was instructed to imagine attempting to move the head forward while the hand provided resistance. Finally, with both hands interlocked behind the head, the patient was instructed to imagine attempting to move the head backward while resisting the movement with the hands. Isometric contractions in all directions were maintained for 5 seconds and performed for 5 repetitions.

**2.2.4 Motor imagery–based stabilization exercises**

Purpose: Purpose: To activate and strengthen the deep neck muscles

Firstly, as a stabilization exercise, a theraband-assisted chin-tuck (cervical flexion) exercise was performed. Participants were seated upright with the head in a neutral position and facing forward. The theraband was placed across the forehead and gently pulled forward by the participant, while the participant was instructed to imagine drawing the chin toward the neck and slightly flexing the head. The position was maintained for 5 seconds before returning to the starting position in a controlled manner. The movement was performed with motor imagery and repeated 5 times.

Secondly, stabilization exercises were performed using motor imagery in a quadruped position. Participants were instructed to imagine a blue mat placed on the floor and to visualize themselves moving onto the mat on their hands and knees. They were asked to imagine the contact of their hands and knees with the mat. First, they imagined performing the cat–camel movement: inhaling and arching the back upward like a cat, holding briefly, and then returning the spine to a neutral position. Next, with the head facing forward in a neutral position, they imagined extending one arm forward and holding the position for 5 seconds, then returning the hand to the mat and repeating the movement with the opposite arm. Subsequently, while maintaining a neutral head position, they imagined extending one leg backward, holding for 5 seconds, then returning the knee to the mat and repeating the movement with the opposite leg. All positions were maintained for 5 seconds and each movement was repeated 5 times.
