# Supplementary material for: Differential changes in cervical proprioception and motor imagery following structured imagery training in chronic neck pain
Source: PLoS One. 2026 Nov 5;21(11):e0361409. doi: 10.1371/journal.pone.0361409 (PMC13739144; doi:10.1371/journal.pone.0361409)
Supplement: Supplementary file 4 [file pone.0361409.s005.docx]

**Academic Editor & Journal Requirements**

Dear Academic Editor,

Thank you for providing us with the opportunity to have our manuscript re-evaluated and for your positive assessment regarding its clinical relevance and conceptual framework. We have carefully considered all comments and suggestions provided by the reviewers. In light of the critical feedback from the statistical reviewer regarding our methodology and data analysis, we have thoroughly revised our statistical analyses, performed the necessary additional evaluations, and updated the Methods, Results, and Discussion sections accordingly.

Our point-by-point responses to each of the academic editor's and reviewers' comments are provided below. All modifications made to the manuscript have been highlighted in the track-changes version, and a clean version without tracking marks has also been prepared and submitted.

**Journal Requirements Response**

**Requirements 1:**

1. Please ensure that your manuscript meets PLOS ONE's style requirements, including those for file naming. The PLOS ONE style templates can be found at

https://journals.plos.org/plosone/s/file?id=wjVg/PLOSOne_formatting_sample_main_body.pdf and 
https://journals.plos.org/plosone/s/file?id=ba62/PLOSOne_formatting_sample_title_authors_affiliations.pdf.

**Response 1:**

Thank you for this reminder. The manuscript has been revised in accordance with the PLOS ONE formatting templates for the main text, title page, author information, and affiliations. The file names and file types have also been prepared in accordance with the journal’s submission requirements.

**Requirements 2:** Thank you for stating the following financial disclosure:

“AB received funding from The Scientific and Technological Research Council of Türkiye (TÜBİTAK) under the 1002 – Short Term R&D Funding Program (grant number 223S175). The funder's website is https://www.tubitak.gov.tr/.
AB: Aysegul Bostan”
       
Please state what role the funders took in the study.  If the funders had no role, please state: "The funders had no role in study design, data collection and analysis, decision to publish, or preparation of the manuscript." 
If this statement is not correct you must amend it as needed. 
Please include this amended Role of Funder statement in your cover letter; we will change the online submission form on your behalf.

**Response 2:**

Thank you for this clarification. The requested Role of the Funder statement has been added to the revised cover letter. The funding was used to cover the costs of materials required for the study and to reimburse participants for travel and meal expenses. The funders had no role in study design, data collection and analysis, decision to publish, or preparation of the manuscript.

**Requirement 3:** In the online submission form you indicate that your data is not available for proprietary reasons and have provided a contact point for accessing this data. Please note that your current contact point is a co-author on this manuscript. According to our Data Policy, the contact point must not be an author on the manuscript and must be an institutional contact, ideally not an individual. Please revise your data statement to a non-author institutional point of contact, such as a data access or ethics committee, and send this to us via return email. Please also include contact information for the third party organization, and please include the full citation of where the data can be found.

**Response 3:**

Thank you for bringing this to our attention and for providing clear guidance regarding the journal's Data Policy. We have revised our Data Availability Statement to specify a non-author, institutional point of contact—specifically, the institutional ethics committee that evaluated and approved the study protocol.

Below is the updated institutional contact information and full data citation, which has also been updated in the online submission portal and the manuscript text:

**Updated Data Availability Statement:**

"The datasets analyzed during the current study are not publicly available due to participant privacy and informed consent restrictions. De-identified data are available upon reasonable request from the Bahçeşehir University Non-Interventional Research Ethics Committee for scientific research purposes.

**Institutional Contact Information:**

- **Entity:** Bahçeşehir University Non-Interventional Research Ethics Committee
- **Contact Person / Office:** Nurcan Vatansever (Coordinator)
- **Email:** nurcan.vatansever@bau.edu.tr
- **Phone:** +90 (216) 579 82 10
- **Address:** Çırağan Caddesi, Osmanpaşa Mektebi Sokak, No: 4-6, 34353 Beşiktaş, Istanbul, Turkey

**Full Data Citation:**

Dataset for the study: "Investigation of the Effects of Lateralization and Motor Imagery Training on EEG Brain Oscillations, Pain Intensity, Proprioception, Kinesiophobia, and Body Awareness in Individuals with Chronic Neck Pain", Bahçeşehir University Non-Interventional Research Ethics Committee, Ethics Approval Decision No: **2023-17/06** (Document No: **E-78097791-020-5621**; Initial Approval Decision No: **2023-09/01**, Dated: **05.03.2023**)."

**Requirement 4:**
Please be informed that funding information should not appear in the Acknowledgments section or other areas of your manuscript. We will only publish funding information present in the Funding Statement section of the online submission form. Please remove any funding-related text from the manuscript.

**Response 4:**

Thank you for this direction. As requested, all funding-related information (including the "Funding" section at lines 600–602) has been completely removed from the manuscript file. The funding details regarding the TÜBİTAK grant (1002 program, grant number 223S175) have been entered solely into the "Funding Statement" section of the online submission portal.

**Requirement 5:**

Please include captions for your Supporting Information files at the end of your manuscript, and update any in-text citations to match accordingly. Please see our Supporting Information guidelines for more information: http://journals.plos.org/plosone/s/supporting-information.

**Response 5:**

Thank you for this clarification. The detailed description of the structured imagery training program and the corresponding exercise have been prepared as a Supporting Information file and labeled “S1 Appendix.” A corresponding in-text citation has been added to the Methods section, and the caption for S1 Appendix has been included at the end of the revised manuscript. The participant flow diagram remains part of the main manuscript as Fig 1 and is cited accordingly.

**Requirement 6:**

If the reviewer comments include a recommendation to cite specific previously published works, please review and evaluate these publications to determine whether they are relevant and should be cited. There is no requirement to cite these works unless the editor has indicated otherwise.

**Response 6:**

Thank you for this clarification. The reviewers did not recommend citing any specific previously published works; therefore, no further action was required regarding this point.

**Additional Editor Comments,**

Dear Authors,

I have received the peer review reports for your manuscript. While two reviewers have evaluated the clinical relevance and conceptual framework of your study favorably (suggesting acceptance and minor revisions), our statistical reviewer has raised major concerns regarding the current methodology/data analysis. Given the importance of statistical rigor for publication, I would like to invite you to submit a revision. I believe your work addresses an important topic in this field, and I look forward to receiving your revised manuscript.

**Response to the Academic Editor:**

Dear Academic Editor,

Thank you for recognizing the clinical relevance and conceptual contribution of our study and for providing us with the opportunity to submit a revised manuscript. We sincerely appreciate your constructive guidance.

We carefully examined all comments raised by the statistical reviewer and comprehensively revised the manuscript accordingly. Regarding the sample size calculation, we acknowledge that the parameters used in the original a priori calculation were incorrectly transcribed in the study protocol and that the calculation was not described in sufficient detail. We corrected this reporting error and expanded the description of the original sample size calculation to include all parameters required for independent replication. Accordingly, we submitted a formal correction request to the Ethics Committee. The sample size calculation is also presented more clearly and comprehensively in the Methods section of the revised manuscript.

We further clarified the study design, participant recruitment, losses to follow-up, reasons for exclusion, and final analysis sample. We added standardized mean differences to provide a more informative assessment of baseline characteristics and performed baseline-adjusted ANCOVA models supported by 5,000-replicate group-stratified BCa bootstrap analyses. In addition, Holm adjustments were applied to address the inflation of Type I error associated with multiple comparisons, and both unadjusted and Holm-adjusted *p*-values are now reported.

The Results, Discussion, Conclusion, and Limitations sections were also revised to distinguish clearly between within-group changes and between-group effects and to ensure that the findings are interpreted consistently with the study design.

We believe that these revisions have substantially improved the methodological transparency, statistical rigor, and interpretive accuracy of the manuscript. We thank you again for the opportunity to revise our work and look forward to your further evaluation.

Sincerely,

The Authors

**Reviewer #1**:

**Comments/questions:**

**Major**

**Reviewer #1 – Comment 1:** The sample size calculation is inadequately reported and cannot be independently replicated. The protocol states that the calculation was based on 80% power, an alpha level of 0.08, and an effect size of 0.08. An alpha of 0.08 is unconventional and may be a typographical error, while an effect size of 0.08 is very small and would not ordinarily result in a sample size of only 17 participants per group. Furthermore, it is unclear which primary outcome this effect size relates to. As a result, the calculation is either incorrect or insufficiently described. In addition, recruitment appears to have been set at the minimum required sample size without any allowance for anticipated withdrawals or loss to follow-up, which is not standard practice in clinical studies. This is potentially a fatal flaw of the work.

**RESPONSE 1:**

We thank the reviewer for this important comment. We acknowledge that the sample size calculation was not previously reported in sufficient detail to permit independent replication and that its parameters were incorrectly transcribed into the study protocol. Re-examination of the original sample size records confirmed that the a priori calculation was performed using G*Power version 3.1.9.2 with a repeated-measures ANOVA model evaluating the group × time interaction for two groups and two measurement time points. The calculation assumed a medium interaction effect (Cohen’s *f*=0.25), α=0.05, 80% statistical power, a correlation of 0.50 between repeated measurements, and a nonsphericity correction of 1.00. These parameters yielded a required total sample size of 34 participants (17 per group), with an actual power of 0.807.

Therefore, the alpha level and effect size reported as 0.08 in the study protocol were transcription errors and did not reflect the original calculation. We also acknowledge that the other parameters required to reproduce the calculation were not specified in the protocol. This was a reporting error and did not represent a retrospective change to the sample size target or the conduct of the study. A formal correction request containing the complete calculation parameters was submitted to the Ethics Committee, and the corresponding approval document has been uploaded with the revision files. The calculation has also been reported in full in the revised Methods section.

Because cervical joint position error (JPE) was one of the primary outcomes, we additionally performed supportive outcome-specific calculations using the within-group effect sizes reported by Sezerel and Yüksel (*dz*=0.81 for flexion JPE and *dz*=0.89 for extension JPE). Using a two-tailed paired-means approach with α=0.05 and 80% power, the required numbers of completers per group were 15 and 12, respectively. After allowing for a potential 10% loss, the more conservative flexion-based estimate corresponded to 17 participants per group and was therefore consistent with the original target. These calculations were conducted only as supportive assessments of sample adequacy for the JPE outcomes and did not replace the original a priori calculation (Lines 129-137).

We further acknowledge that participant recruitment and attrition were not described sufficiently clearly in the previous version. Recruitment was not stopped when 34 individuals had merely been enrolled. Rather, the target established by the sample size calculation was 34 participants—17 per group—with complete and analyzable data. Although a fixed attrition percentage was not specified in the protocol, recruitment continued until this target was reached.

A total of 39 participants were enrolled in the study, five of whom were not included in the final analysis. One participant in the HC group withdrew consent after the baseline assessment, two participants in the CNP group did not complete the intervention program, and data from two additional participants in the CNP group could not be analyzed because of technical or data-quality issues. Consequently, the final analyses included the prespecified target of 34 participants, with 17 participants in each group.

The participant flow and the reasons for withdrawal, non-completion, and exclusion have now been described in detail in the revised Methods section and participant flow diagram. We acknowledge that the previous version did not clearly distinguish between the number of participants enrolled and the target number of participants with complete and analyzable data, which may have led to misunderstanding. In response to the reviewer’s comment, we have revised the manuscript to provide a reproducible description of the sample size calculation and a transparent account of recruitment, attrition, and the final analytical sample (Lines 137–142).

**Reviewer #1 – Comment 2:** The interpretation relies heavily on p-values despite uncertainty around the sample size calculation and limited statistical power. Several conclusions are based on statistically significant within-group changes, whereas the more informative between-group comparisons are often non-significant. Given the small sample size and concerns regarding the power calculation, greater emphasis should be placed on effect sizes and confidence intervals rather than statistical significance alone. Analysing using a regression framework, would allow for that.

**RESPONSE 2**

We sincerely thank the reviewer for this important methodological comment and agree that unadjusted within-group *p*-values should not form the primary basis for interpreting the findings. In accordance with the reviewer’s recommendation, we substantially revised the statistical analysis and reporting by applying baseline-adjusted regression/ANCOVA models to the post-training outcomes. The results are now presented primarily in terms of adjusted between-group differences, effect sizes (partial η²), and 95% bias-corrected and accelerated (BCa) confidence intervals obtained from 5,000 group-stratified bootstrap resamples (Tables 6 and 8). We also applied the Holm correction to address multiple comparisons and reported both unadjusted and Holm-adjusted *p*-values.

The revised analyses showed that the unadjusted within-group reduction in extension JPE observed in the CNP group was not supported by the between-group analyses. The baseline-adjusted difference was −0.462° (BCa 95% CI: −2.041 to 1.069; unadjusted bootstrap *p*=0.597; Holm-adjusted *p*=1.000; partial η²=0.009), providing no evidence of a differential training response. In contrast, the CNP group continued to demonstrate higher post-training flexion JPE than the HC group after adjustment for baseline values (adjusted difference=3.316°, BCa 95% CI: 2.008 to 4.743; unadjusted bootstrap *p*=0.001; Holm-adjusted *p*=0.016; partial η²=0.310). This finding indicates persistence of the between-group difference in flexion JPE rather than an effect of training.

Accordingly, we removed our previous claim that SIT produced a selective improvement in cervical proprioception. The Results and Discussion sections have been comprehensively revised to distinguish unadjusted within-group findings from baseline-adjusted between-group results and to place greater emphasis on adjusted estimates, confidence intervals, and effect sizes. Given the sample size, non-significant findings have not been interpreted as evidence of equivalence, and the conclusions have been revised to provide a more cautious interpretation of the results.

**Reviewer #1 – Comment 3:** Baseline demographic and cognitive characteristics appear broadly balanced between groups and are appropriately reported. However, given the small sample size, reliance on non-significant p-values alone is insufficient to demonstrate balance, and important baseline differences were present for several outcome measures. SMDs would demonstrate that. A regression framework could potentially address that, although the sample is pretty small and models may be unstable.

**RESPONSE 3:**

We thank the reviewer for this valuable comment. We agree that, particularly in a small sample, non-significant *p*-values alone are insufficient to demonstrate baseline balance. Therefore, standardized mean differences (SMDs) were calculated for all baseline demographic, cognitive, and motor imagery variables that permitted between-group comparison and were added to Tables 2 and 3. The absolute SMDs ranged from 0.000 to 0.462 for demographic characteristics and from 0.027 to 0.337 for cognitive and motor imagery measures. Reporting these estimates allows the magnitude of baseline differences to be evaluated independently of statistical significance. The Statistical Analysis and Results sections were revised accordingly, and baseline characteristics are now interpreted using both p-values and SMDs.

To account for baseline differences in outcomes assessed in both groups, separate baseline-adjusted linear models (ANCOVA) were fitted for each eligible outcome. In each model, the post-training value was entered as the dependent variable, group as the fixed factor, and the corresponding baseline value as the sole covariate. No additional covariates were included to reduce the risk of overfitting and model instability associated with the small sample. Adjusted group means and between-group differences were reported together with partial η² values and 95% bias-corrected and accelerated (BCa) confidence intervals obtained from 5,000 group-stratified bootstrap resamples (Tables 6 and 8). The potential instability of the estimates due to the small sample size has been acknowledged, and the results have been interpreted cautiously.

VAS, TSK, and NDI were assessed only in the CNP group. Because corresponding baseline and post-training measurements were unavailable for the HC group, baseline-adjusted between-group ANCOVA models could not be fitted for these outcomes. They were therefore reported as pre–post changes within the CNP group and were not included in the between-group ANCOVA tables. The multiplicity adjustment applied to these within-group clinical comparisons is described in our response to Comment 4.

Although FreNAQ-T was assessed in both groups, the baseline-adjusted model for this outcome was not considered sufficiently reliable because of the marked floor effect in the HC group, limited overlap between the groups’ baseline distributions, and substantial heterogeneity of error variances (Levene’s test, *p*<0.001). Baseline-adjusted FreNAQ-T estimates were therefore not included in the primary ANCOVA results. Instead, the raw values and change scores were reported in Table 4, and the within-CNP pre–post comparison was included in the Holm correction applied to the within-group clinical outcomes. Because a reliable baseline-adjusted between-group estimate could not be obtained, the FreNAQ-T finding was interpreted as exploratory and with appropriate caution.

Accordingly, the revised manuscript now evaluates baseline characteristics using SMDs, applies baseline-adjusted models to eligible outcomes measured in both groups, and explains why outcomes unsuitable for between-group ANCOVA were analyzed and reported differently. These revisions are described in the Statistical Analysis and Results sections (Lines 251-72; 300–55; 379-88).

**Reviewer #1 – Comment 4:** there are numerous outcomes tested in a small sample - the risk of type-I error increase is very large.

**RESPONSE 4**

We thank the reviewer for highlighting the increased risk of Type I error associated with testing numerous outcomes in a relatively small sample. We agree that multiplicity was not adequately addressed in the original analysis.

In response, we applied the Holm step-down procedure within two inferential families defined according to the analytical questions addressed:

1. The 16 baseline-adjusted between-group comparisons comprising the clinical, sensorimotor, cognitive, and laterality outcomes reported in Tables 6 and 8; and
2. The four within-group clinical pre–post comparisons evaluated in the CNP group (VAS, TSK, NDI, and FreNAQ-T; Table 4).

For the first family, the Holm procedure was applied jointly to the 16 unadjusted bootstrap *p*-values obtained from the baseline-adjusted between-group models in Tables 6 and 8. Following correction, only the difference in post-training flexion JPE remained statistically significant (unadjusted bootstrap *p*=0.001; Holm-adjusted *p*=0.016). The unadjusted difference in sagittal-plane JPE (*p*=0.041) did not remain significant after correction (Holm-adjusted *p*=0.615), and the Holm-adjusted *p*-values for the remaining outcomes were 1.000. Both unadjusted and Holm-adjusted *p*-values are now reported in Tables 6 and 8.

For the second family, the Holm procedure was applied across the four within-CNP clinical pre–post comparisons. The Holm-adjusted *p*-values were 0.012 for VAS, 0.034 for TSK, 0.034 for NDI, and 0.012 for FreNAQ-T; all four remained statistically significant after correction. These outcomes were treated as a separate family because they addressed a distinct within-group clinical question. VAS, TSK, and NDI could not be included in the baseline-adjusted between-group analyses because corresponding measurements were not available for the HC group. Although FreNAQ-T was assessed in both groups, a reliable baseline-adjusted between-group model could not be obtained because of the marked floor effect in the HC group, limited overlap in baseline distributions, and substantial variance heterogeneity. Its within-CNP comparison was therefore included with the other clinical pre–post outcomes and interpreted cautiously.

All other unadjusted within-group and between-group comparisons are presented descriptively and are not used as the primary basis for inference. The two outcome families and the multiplicity-adjustment strategy were not prespecified in the study protocol but were introduced during the revision in response to the reviewer’s concern. This has been stated transparently, and the findings have been interpreted cautiously. The Statistical Analysis, Results, Discussion, and Limitations sections have been revised accordingly.

**Minor**
**Reviewer #1 – Comment 5:** clarify if all the data were complete for all participants, in the methods section, as seems to be the case.

**RESPONSE 5:**

We thank the reviewer for the opportunity to clarify data completeness. A total of 39 participants were enrolled: 21 in the CNP group and 18 in the HC group. One HC participant withdrew consent after the baseline assessment, two CNP participants discontinued the intervention, and two additional CNP participants were excluded because their data were not analyzable owing to technical or data-quality issues. Consequently, the final analytical sample comprised 34 participants, with 17 participants in each group.

For these 34 participants, the dataset was complete for all outcomes applicable to their respective groups and included in the reported analyses; therefore, no missing-data imputation was performed. VAS, TSK, and NDI were collected only in the CNP group by design and were not considered missing for the HC group. The Methods section and participant flow diagram have been revised to clarify participant losses, exclusions, the final analytical sample, and data completeness (Lines 137–142; Fig 1).

**Reviewer #2:**

Dear authors

Congratulations
Overall, this study provides novel information regarding the differential responses of peripheral and central mechanisms following structured imagery training. The manuscript is written in clear scientific English and is supported by relevant and up-to-date literature. The conclusions are consistent with the presented results and have potential implications for future rehabilitation research and clinical practice.

I appreciate the authors' efforts and believe that this manuscript makes a meaningful contribution to the field. I have no major concerns and recommend its acceptance for publication.

Best regards.

**Response to Reviewer #2**

We sincerely thank the reviewer for the positive and encouraging evaluation of our manuscript. We greatly appreciate the reviewer’s recognition of the novelty, scientific clarity, and potential contribution of our study to rehabilitation research and clinical practice. We are grateful for the reviewer’s thoughtful comments and favorable recommendation.

**Comments/questions:**

**Reviewer #3:** This manuscript addresses an interesting and clinically relevant topic by investigating the effects of structured imagery training on cervical proprioception, clinical outcomes, and implicit motor imagery in individuals with chronic neck pain. The simultaneous assessment of proprioceptive function and motor imagery performance is a valuable aspect of the study. The manuscript is generally well presented; however, several points regarding the interpretation of the findings and the description of the study design would benefit from clarification.

**Reviewer #3 – Comment 1:** Interpretation of the primary outcome. The primary outcome findings are clearly reported in the Results, including the non-significant between-group differences in JPE change scores. However, the conclusion that SIT “selectively enhanced CP” appears somewhat stronger than supported by these findings. Although extension JPE significantly improved within the CNP group (p=0.039), the between-group difference in change scores did not reach statistical significance (p=0.084). Similarly, the between-group difference in flexion JPE change was not significant (p=0.998).
Therefore, the authors are encouraged to distinguish more clearly between within-group improvement and between-group effects when interpreting the findings and to reconsider statements suggesting that SIT selectively enhanced cervical proprioception. The proposed dissociation between peripheral and central mechanisms should also be presented more cautiously in light of these findings.

**RESPONSE 1:**

We thank the reviewer for this important observation. We agree that our previous statement that SIT “selectively enhanced cervical proprioception” was stronger than warranted by the findings and did not adequately distinguish within-group change from between-group effects.

As noted by the reviewer, extension JPE decreased within the CNP group (unadjusted *p*=0.039); however, the between-group difference in extension JPE change did not reach statistical significance (*p*=0.084). Similarly, no significant between-group difference was observed in flexion JPE change (*p*=0.998). Therefore, the unadjusted within-group reduction in extension JPE cannot be interpreted as evidence that the CNP group demonstrated a differential response to training compared with the HC group.

To account for baseline differences, we additionally fitted ANCOVA models in which the post-training JPE value was entered as the dependent variable, group as the fixed factor, and the corresponding baseline JPE value as the covariate. For extension JPE, the baseline-adjusted post-training difference was not statistically significant (Holm-adjusted *p*=1.000), consistent with the absence of a significant between-group difference in change. For flexion JPE, the CNP group continued to demonstrate a higher baseline-adjusted post-training value than the HC group (Holm-adjusted *p*=0.016). This finding indicates that the pre-existing difference in flexion JPE persisted after training; it does not demonstrate a differential effect of SIT.

Accordingly, the statement that SIT “selectively enhanced cervical proprioception” has been removed from the Abstract, Discussion, and Conclusion. The revised manuscript now clearly distinguishes the unadjusted within-group reduction in extension JPE from the non-significant between-group differences in JPE change. We have also clarified that, because both groups received the same training and the study did not include an untreated CNP control group, the observed within-group changes cannot be attributed exclusively to SIT.

We have also removed statements presenting the findings as evidence of a dissociation between peripheral and central mechanisms. The differing patterns observed across JPE, laterality, and cognitive measures are now discussed cautiously as potentially reflecting distinct measurement domains rather than as evidence of a specific neural mechanism. Because the study did not directly assess the underlying neural processes, no conclusions regarding central compensation or cortical reorganization are drawn. The relevant sections of the Abstract, Results, Discussion, Limitations, and Conclusion have been revised accordingly.

**Reviewer #3 – Comment 2:** Multiple comparisons

Given the number of statistical comparisons conducted across the primary and secondary outcomes, it would be helpful for the authors to clarify whether the potential inflation of Type I error due to multiple testing was considered. If no adjustment for multiple comparisons was applied, this should be stated, and the secondary findings, particularly those with p-values close to 0.05, should be interpreted cautiously.

**RESPONSE 2:**

We thank the reviewer for highlighting the potential inflation of Type I error due to multiple comparisons. We agree that multiplicity was not adequately addressed in the original analysis.

In response, we applied the Holm step-down correction separately within two inferential families: (1) the 16 baseline-adjusted between-group comparisons comprising the clinical, sensorimotor, cognitive, and laterality outcomes reported in Tables 6 and 8; and (2) the four within-group clinical pre–post comparisons evaluated in the CNP group (VAS, TSK, NDI, and FreNAQ-T; Table 4).

For the first family, the Holm procedure was applied jointly to the 16 unadjusted bootstrap *p*-values reported in Tables 6 and 8. Both unadjusted and Holm-adjusted *p*-values are now presented in these tables. Following correction, only the baseline-adjusted post-training difference in flexion JPE remained statistically significant (Holm-adjusted *p*=0.016). The unadjusted difference in sagittal-plane JPE (*p*=0.041) did not remain significant after correction (Holm-adjusted *p*=0.615), and none of the baseline-adjusted laterality comparisons were statistically significant.

For the second family, the Holm procedure was applied across the four within-CNP clinical comparisons. The Holm-adjusted *p*-values were 0.012 for VAS, 0.034 for TSK, 0.034 for NDI, and 0.012 for FreNAQ-T. These values are presented in parentheses alongside the corresponding unadjusted *p*-values in Table 4. The remaining unadjusted within-group and between-group comparisons are presented descriptively and are not used as the primary basis for inference.

Because the outcome families and multiplicity-adjustment strategy were introduced during the revision and were not prespecified in the study protocol, this has been stated transparently, and findings based on unadjusted comparisons—particularly those with *p*-values close to 0.05—are interpreted cautiously and as exploratory. The Statistical Analysis, Results, Discussion, and Limitations sections have been revised accordingly

**Reviewer #3 – Comment 3:** Clarification and consistency of the study design

The description of the study design would benefit from greater consistency throughout the manuscript. In the Methods, the study is described as a “two-group controlled clinical trial” with reference to CONSORT, whereas the limitations section refers to a “quasi-experimental design” and recommends randomized designs for future research.
The authors are kindly asked to clarify whether random allocation was performed and to use consistent terminology when describing the study design throughout the manuscript.

**RESPONSE 3:**

We thank the reviewer for identifying the inconsistency in the description of the study design. We confirm that no random allocation was performed. Participants were included in either the CNP group or the HC group based on their clinical status and the relevant eligibility criteria, and both groups received the same structured imagery training program.

Accordingly, the study is now consistently described throughout the manuscript as a “prospective, non-randomized, two-group pre–post quasi-experimental design.” The previous description of the study as a “two-group controlled clinical trial,” together with any wording that could imply random allocation, has been removed. The reference to CONSORT has also been removed to avoid presenting the study as a randomized controlled trial.

The Study Design section now explicitly states that participants were classified into groups according to their clinical status and that no randomization was performed. The Abstract, Methods, Discussion, and Limitations sections have been reviewed and revised to ensure consistent terminology. The participant flow diagram (Fig 1) has also been updated to clearly present participant recruitment, group classification, follow-up, and analysis (Lines 137–142).

**Reviewer #3 – Comment 4:**  Interpretation of the control condition

Both the CNP and healthy control groups received the structured imagery training. Therefore, the present design primarily compares the responses of individuals with CNP and healthy participants to the same intervention rather than evaluating the efficacy of SIT against an untreated CNP control condition. The authors are encouraged to acknowledge this distinction more explicitly when discussing intervention efficacy and to use causal terminology cautiously throughout the manuscript.

The study addresses a relevant research question and is generally well presented. However, clarification of the study design and a more cautious interpretation of the primary outcome and intervention effects would improve the accuracy and clarity of the manuscript.

**RESPONSE 4:**

We thank the reviewer for highlighting this important distinction. We agree that, because both the CNP and HC groups received the same structured imagery training and the study did not include an untreated CNP control group, the present design does not allow the efficacy of SIT to be established against a no-intervention condition.

We have therefore clarified that the study primarily compares the pre–post response patterns of individuals with CNP and healthy participants following the same training program. Although within-group changes were observed in several clinical outcomes in the CNP group, these changes cannot be attributed exclusively to SIT, as they may also reflect natural variation, repeated testing, participant expectations, or other nonspecific effects. Furthermore, a statistically significant change within one group accompanied by a non-significant change within the other was not interpreted as evidence of a differential response unless supported by an appropriate between-group or baseline-adjusted comparison.

Accordingly, statements implying that SIT definitively caused the observed changes or established intervention efficacy have been removed or revised. Expressions such as “SIT improved,” “SIT enhanced,” and “the effect of SIT” have been replaced, where appropriate, with descriptive wording such as “within-group reductions were observed after training,” “post-training changes were observed,” and “the groups’ responses following the same training program were compared.”

This distinction has been explicitly addressed in the study aim, Discussion, and Limitations sections. The Conclusion has also been revised to summarize the observed findings descriptively without making causal claims regarding SIT. The absence of an untreated CNP control group is now acknowledged as an important design limitation, and future randomized controlled studies including an untreated, usual-care, or active-comparator CNP group are recommended to evaluate the efficacy of SIT more directly.
