# Supplementary material for: Differential changes in cervical proprioception and motor imagery following structured imagery training in chronic neck pain
Source: PLoS One. 2026 Nov 5;21(11):e0361409. doi: 10.1371/journal.pone.0361409 (PMC13739144; doi:10.1371/journal.pone.0361409)
Supplement: Peer review history

# Peer review: Decision Letter 0

**DOI:** 10.1371/journal.pone.0361409.r001

**Authors:** Fatih Özden

24 Aug 2026

Dear Dr. Bostan,

Thank you for submitting your manuscript to PLOS One. After careful consideration, we feel that it has merit but does not fully meet PLOS One’s publication criteria as it currently stands. Therefore, we invite you to submit a revised version of the manuscript that addresses the points raised during the review process.

Dear Authors, I have received the peer review reports for your manuscript. While two reviewers have evaluated the clinical relevance and conceptual framework of your study favorably (suggesting acceptance and minor revisions), our statistical reviewer has raised major concerns regarding the current methodology/data analysis. Given the importance of statistical rigor for publication, I would like to invite you to submit a revision. I believe your work addresses an important topic in this field, and I look forward to receiving your revised manuscript.

Please submit your revised manuscript by Oct 23 2026 11:59PM. If you will need more time than this to complete your revisions, please reply to this message or contact the journal office at plosone@plos.org. When you're ready to submit your revision, log on to https://www.editorialmanager.com/pone/ and select the 'Submissions Needing Revision' folder to locate your manuscript file.

- A letter that responds to each point raised by the academic editor and reviewer(s). You should upload this letter as a separate file labeled 'Response to Reviewers'.
- A marked-up copy of your manuscript that highlights changes made to the original version. You should upload this as a separate file labeled 'Revised Manuscript with Track Changes'.
- An unmarked version of your revised paper without tracked changes. You should upload this as a separate file labeled 'Manuscript'.

If you would like to make changes to your financial disclosure, please include your updated statement in your cover letter. Guidelines for resubmitting your figure files are available below the reviewer comments at the end of this letter.

If applicable, we recommend that you deposit your laboratory protocols in protocols.io to enhance the reproducibility of your results. Protocols.io assigns your protocol its own identifier (DOI) so that it can be cited independently in the future. For instructions see: https://journals.plos.org/plosone/s/submission-guidelines#loc-laboratory-protocols. Additionally, PLOS One offers an option for publishing peer-reviewed Lab Protocol articles, which describe protocols hosted on protocols.io. Read more information on sharing protocols at https://plos.org/protocols?utm_medium=editorial-email&utm_source=authorletters&utm_campaign=protocols.

As the corresponding author, your ORCID iD is verified in the submission system and will appear in the published article. PLOS supports the use of ORCID, and we encourage all coauthors to register for an ORCID iD and use it as well. Please encourage your coauthors to verify their ORCID iD within the submission system before final acceptance, as unverified ORCID iDs will not appear in the published article. *Only*  the individual author can complete the verification step; PLOS staff *cannot*  verify ORCID iDs on behalf of authors.

We look forward to receiving your revised manuscript.

Kind regards,

Fatih Özden, PT, PhD, Assoc. Prof,

Academic Editor

PLOS One

Journal Requirements:

When submitting your revision, we need you to address these additional requirements.

1. Please ensure that your manuscript meets PLOS ONE's style requirements, including those for file naming. The PLOS ONE style templates can be found at

https://journals.plos.org/plosone/s/file?id=wjVg/PLOSOne_formatting_sample_main_body.pdf and

https://journals.plos.org/plosone/s/file?id=ba62/PLOSOne_formatting_sample_title_authors_affiliations.pdf.

2. Thank you for stating the following financial disclosure:

“AB received funding from The Scientific and Technological Research Council of Türkiye (TÜBİTAK) under the 1002 – Short Term R&D Funding Program (grant number 223S175). The funder's website is https://www.tubitak.gov.tr/.

AB: Aysegul Bostan”

Please state what role the funders took in the study.  If the funders had no role, please state: "The funders had no role in study design, data collection and analysis, decision to publish, or preparation of the manuscript."

If this statement is not correct you must amend it as needed.

Please include this amended Role of Funder statement in your cover letter; we will change the online submission form on your behalf.

3. In the online submission form you indicate that your data is not available for proprietary reasons and have provided a contact point for accessing this data. Please note that your current contact point is a co-author on this manuscript. According to our Data Policy, the contact point must not be an author on the manuscript and must be an institutional contact, ideally not an individual. Please revise your data statement to a non-author institutional point of contact, such as a data access or ethics committee, and send this to us via return email. Please also include contact information for the third party organization, and please include the full citation of where the data can be found.

4. Please be informed that funding information should not appear in the Acknowledgments section or other areas of your manuscript. We will only publish funding information present in the Funding Statement section of the online submission form. Please remove any funding-related text from the manuscript.

5. Please include captions for your Supporting Information files at the end of your manuscript, and update any in-text citations to match accordingly. Please see our Supporting Information guidelines for more information: http://journals.plos.org/plosone/s/supporting-information.

6. If the reviewer comments include a recommendation to cite specific previously published works, please review and evaluate these publications to determine whether they are relevant and should be cited. There is no requirement to cite these works unless the editor has indicated otherwise.

Additional Editor Comments (if provided):

Dear Authors, I have received the peer review reports for your manuscript. While two reviewers have evaluated the clinical relevance and conceptual framework of your study favorably (suggesting acceptance and minor revisions), our statistical reviewer has raised major concerns regarding the current methodology/data analysis. Given the importance of statistical rigor for publication, I would like to invite you to submit a revision. I believe your work addresses an important topic in this field, and I look forward to receiving your revised manuscript.

[Note: HTML markup is below. Please do not edit.]

Reviewers' comments:

Reviewer's Responses to Questions

**Comments to the Author**

1. Is the manuscript technically sound, and do the data support the conclusions?

Reviewer #1: Partly

Reviewer #2: Yes

Reviewer #3: Yes

**********

2. Has the statistical analysis been performed appropriately and rigorously?

Reviewer #1: Yes

Reviewer #2: Yes

Reviewer #3: Yes

**********

3. Have the authors made all data underlying the findings in their manuscript fully available?

The PLOS Data policy

Reviewer #1: Yes

Reviewer #2: Yes

Reviewer #3: No

**********

4. Is the manuscript presented in an intelligible fashion and written in standard English?

Reviewer #1: Yes

Reviewer #2: No

Reviewer #3: Yes

**********

Reviewer #1: As the statistical reviewer I will focus on methods and reporting.

Major

1) The sample size calculation is inadequately reported and cannot be independently replicated. The protocol states that the calculation was based on 80% power, an alpha level of 0.08, and an effect size of 0.08. An alpha of 0.08 is unconventional and may be a typographical error, while an effect size of 0.08 is very small and would not ordinarily result in a sample size of only 17 participants per group. Furthermore, it is unclear which primary outcome this effect size relates to. As a result, the calculation is either incorrect or insufficiently described. In addition, recruitment appears to have been set at the minimum required sample size without any allowance for anticipated withdrawals or loss to follow-up, which is not standard practice in clinical studies. This is potentially a fatal flaw of the work.

2) The interpretation relies heavily on p-values despite uncertainty around the sample size calculation and limited statistical power. Several conclusions are based on statistically significant within-group changes, whereas the more informative between-group comparisons are often non-significant. Given the small sample size and concerns regarding the power calculation, greater emphasis should be placed on effect sizes and confidence intervals rather than statistical significance alone. Analysing using a regression framework, would allow for that.

3) Baseline demographic and cognitive characteristics appear broadly balanced between groups and are appropriately reported. However, given the small sample size, reliance on non-significant p-values alone is insufficient to demonstrate balance, and important baseline differences were present for several outcome measures. SMDs would demonstrate that. A regression framework could potentially address that, although the sample is pretty small and models may be unstable.

4) there are numerous outcomes tested in a small sample - the risk of type-I error increase is very large.

Minor

1) clarify if all the data were complete for all participants, in the methods section, as seems to be the case.

Reviewer #2: Dear authors

Congratulations

Overall, this study provides novel information regarding the differential responses of peripheral and central mechanisms following structured imagery training. The manuscript is written in clear scientific English and is supported by relevant and up-to-date literature. The conclusions are consistent with the presented results and have potential implications for future rehabilitation research and clinical practice.

I appreciate the authors' efforts and believe that this manuscript makes a meaningful contribution to the field. I have no major concerns and recommend its acceptance for publication.

Best regards.

Reviewer #3: This manuscript addresses an interesting and clinically relevant topic by investigating the effects of structured imagery training on cervical proprioception, clinical outcomes, and implicit motor imagery in individuals with chronic neck pain. The simultaneous assessment of proprioceptive function and motor imagery performance is a valuable aspect of the study. The manuscript is generally well presented; however, several points regarding the interpretation of the findings and the description of the study design would benefit from clarification.

Comments

1. Interpretation of the primary outcome

The primary outcome findings are clearly reported in the Results, including the non-significant between-group differences in JPE change scores. However, the conclusion that SIT “selectively enhanced CP” appears somewhat stronger than supported by these findings. Although extension JPE significantly improved within the CNP group (p=0.039), the between-group difference in change scores did not reach statistical significance (p=0.084). Similarly, the between-group difference in flexion JPE change was not significant (p=0.998).

Therefore, the authors are encouraged to distinguish more clearly between within-group improvement and between-group effects when interpreting the findings and to reconsider statements suggesting that SIT selectively enhanced cervical proprioception. The proposed dissociation between peripheral and central mechanisms should also be presented more cautiously in light of these findings.

2. Multiple comparisons

Given the number of statistical comparisons conducted across the primary and secondary outcomes, it would be helpful for the authors to clarify whether the potential inflation of Type I error due to multiple testing was considered. If no adjustment for multiple comparisons was applied, this should be stated, and the secondary findings, particularly those with p-values close to 0.05, should be interpreted cautiously.

3. Clarification and consistency of the study design

The description of the study design would benefit from greater consistency throughout the manuscript. In the Methods, the study is described as a “two-group controlled clinical trial” with reference to CONSORT, whereas the limitations section refers to a “quasi-experimental design” and recommends randomized designs for future research.

The authors are kindly asked to clarify whether random allocation was performed and to use consistent terminology when describing the study design throughout the manuscript.

4. Interpretation of the control condition

Both the CNP and healthy control groups received the structured imagery training. Therefore, the present design primarily compares the responses of individuals with CNP and healthy participants to the same intervention rather than evaluating the efficacy of SIT against an untreated CNP control condition.

The authors are encouraged to acknowledge this distinction more explicitly when discussing intervention efficacy and to use causal terminology cautiously throughout the manuscript.

The study addresses a relevant research question and is generally well presented. However, clarification of the study design and a more cautious interpretation of the primary outcome and intervention effects would improve the accuracy and clarity of the manuscript.

**********

what does this mean?). If published, this will include your full peer review and any attached files.

If you choose “no”, your identity will remain anonymous but your review may still be made public.

**Do you want your identity to be public for this peer review?**  For information about this choice, including consent withdrawal, please see our Privacy Policy

Reviewer #1: No

Reviewer #2: **Yes:**  Mehmet Özkeskin

Reviewer #3: No

**********

[NOTE: If reviewer comments were submitted as an attachment file, they will be attached to this email and accessible via the submission site. Please log into your account, locate the manuscript record, and check for the action link "View Attachments". If this link does not appear, there are no attachment files.]

To ensure your figures meet our technical requirements, please review our figure guidelines: https://journals.plos.org/plosone/s/figures

You may also use PLOS’s free figure tool, NAAS, to help you prepare publication quality figures: https://journals.plos.org/plosone/s/figures#loc-tools-for-figure-preparation.

NAAS will assess whether your figures meet our technical requirements by comparing each figure against our figure specifications.

# Author comment: Author response to Decision Letter 1

**DOI:** 10.1371/journal.pone.0361409.r002

14 Sep 2026

We thank the editor and reviewers for their careful evaluation of our manuscript and their constructive comments. We have carefully addressed all comments and revised the manuscript accordingly. A detailed point-by-point response to the editor and reviewers has been uploaded as a separate file titled “Response to Reviewers.”

Attachment Submitted filename: Response to Reviewers.docx

# Peer review: Decision Letter 1

**DOI:** 10.1371/journal.pone.0361409.r003

**Authors:** Hasan Sozen

7 Oct 2026

Dissociation Between Cervical Proprioception and Motor Imagery Following Structured Imagery Training in Chronic Neck Pain

PONE-D-26-27202R1

Dear Dr. Bostan,

We’re pleased to inform you that your manuscript has been judged scientifically suitable for publication and will be formally accepted for publication once it meets all outstanding technical requirements.

Within one week, you’ll receive an e-mail detailing the required amendments. When these have been addressed, you’ll receive a formal acceptance letter and your manuscript will be scheduled for publication.

An invoice will be generated when your article is formally accepted. Please note, if your institution has a publishing partnership with PLOS and your article meets the relevant criteria, all or part of your publication costs will be covered. Please make sure your user information is up-to-date by logging into Editorial Manager at Editorial Manager® and clicking the ‘Update My Information' link at the top of the page. For questions related to billing, please contact billing support.

If your institution or institutions have a press office, please notify them about your upcoming paper to help maximize its impact. If they’ll be preparing press materials, please inform our press team as soon as possible -- no later than 48 hours after receiving the formal acceptance. Your manuscript will remain under strict press embargo until 2 pm Eastern Time on the date of publication. For more information, please contact onepress@plos.org.

Kind regards,

Hasan Sozen

Academic Editor

PLOS One

Additional Editor Comments:

The revised manuscript has been substantially improved in terms of statistical analysis, transparency and interpretation. The authors have correctly distinguished between within-group changes and baseline-adjusted between-group comparisons, and have addressed multiplicity using the Holm correction method. However, a few issues remain. Firstly, the term 'dissociation' in the title is stronger than the revised interpretation warrants, particularly since the study does not directly assess central or peripheral mechanisms. Additionally, causal wording such as 'provided short-term relief' should be softened further because both groups received the same intervention, and there was no control group for untreated CNP. Finally, alongside statistical significance, the clinical relevance of the relatively small changes in VAS, NDI and FreNAQ-T should be discussed. Addressing these points would improve the accuracy and consistency of the manuscript further.

Reviewers' comments:

Reviewer's Responses to Questions

**Comments to the Author**

Reviewer #1: All comments have been addressed

Reviewer #2: All comments have been addressed

Reviewer #3: All comments have been addressed

**********

2. Is the manuscript technically sound, and do the data support the conclusions?

Reviewer #1: Yes

Reviewer #2: Yes

Reviewer #3: Yes

**********

3. Has the statistical analysis been performed appropriately and rigorously?

Reviewer #1: Yes

Reviewer #2: Yes

Reviewer #3: Yes

**********

4. Have the authors made all data underlying the findings in their manuscript fully available?

The PLOS Data policy

Reviewer #1: Yes

Reviewer #2: Yes

Reviewer #3: Yes

**********

5. Is the manuscript presented in an intelligible fashion and written in standard English?

Reviewer #1: Yes

Reviewer #2: Yes

Reviewer #3: Yes

**********

Reviewer #1: I am satisfied with the authors' responses and the resulting changes to the paper. I have nothing else to add.

Reviewer #2: Dear authors,

I thank the authors for addressing all comments and making the requested revisions. I have no further suggestions.

Reviewer #3: I would like to thank the authors for their careful responses to my comments and for the revisions made to the manuscript. The issues raised have been adequately addressed, and the revisions have improved the clarity and scientific quality of the manuscript. I have no further comments.

**********

what does this mean?). If published, this will include your full peer review and any attached files.

If you choose “no”, your identity will remain anonymous but your review may still be made public.

**Do you want your identity to be public for this peer review?**  For information about this choice, including consent withdrawal, please see our Privacy Policy

Reviewer #1: No

Reviewer #2: **Yes:**  Mehmet Özkeskin

Reviewer #3: No

**********

# Editor report: Acceptance letter

**DOI:** 10.1371/journal.pone.0361409.r004

**Authors:** Hasan Sozen

PONE-D-26-27202R1

PLOS One

Dear Dr. Bostan,

I'm pleased to inform you that your manuscript has been deemed suitable for publication in PLOS One. Congratulations! Your manuscript is now being handed over to our production team.

At this stage, our production department will prepare your paper for publication. This includes ensuring the following:

* All references, tables, and figures are properly cited

* All relevant supporting information is included in the manuscript submission,

* There are no issues that prevent the paper from being properly typeset

You will receive further instructions from the production team, including instructions on how to review your proof when it is ready. Please keep in mind that we are working through a large volume of accepted articles, so please give us a few days to review your paper and let you know the next and final steps.

Lastly, if your institution or institutions have a press office, please let them know about your upcoming paper now to help maximize its impact. If they'll be preparing press materials, please inform our press team within the next 48 hours. Your manuscript will remain under strict press embargo until 2 pm Eastern Time on the date of publication. For more information, please contact onepress@plos.org.

You will receive an invoice from PLOS for your publication fee after your manuscript has reached the completed accept phase. If you receive an email requesting payment before acceptance or for any other service, this may be a phishing scheme. Learn how to identify phishing emails and protect your accounts at https://explore.plos.org/phishing.

If we can help with anything else, please email us at customercare@plos.org.

Thank you for submitting your work to PLOS One and supporting open access.

Kind regards,

PLOS One Editorial Office Staff

on behalf of

Assoc. Prof. Hasan Sozen

Academic Editor

PLOS One
